# Silver integrated hybrids and nanocomposites for next-generation biomedicine: Beyond antimicrobial coatings toward smart sense–response–heal platforms

**DOI:** 10.1016/j.mtbio.2025.102609

**Published:** 2025-11-26

**Authors:** Eileen Tabrizi, Bingyun Li

**Affiliations:** a Department of Orthopaedics, School of Medicine, West Virginia University, Morgantown, WV, 26506-9196, USA; b School of Pharmacy, West Virginia University, Morgantown, WV, 26506, USA

**Keywords:** Silver nanocomposite, Multifunctional biomaterial, Data-driven design, Stimuli-responsive nanoplatform, Antimicrobial resistance, Regenerative medicine, Biointeractive composite

## Abstract

Silver has re-emerged as a key material in biomedicine, functioning not only as an antimicrobial coating but also as a programmable nanoscale mediator that links infection control, tissue regeneration, and responsive therapeutics. At nanometer scales, silver's plasmonic, catalytic, and electronic properties differ significantly from those in bulk form. Advances in nanocomposite engineering have transitioned the field from simple antibacterial fillers to multifaceted platforms capable of sensing, signaling, and adapting to complex biological environments. By integrating silver with polymers, carbon allotropes, ceramics, metals, and bioactive scaffolds, researchers have developed silver nanocomposite-based architectures that promote angiogenesis, immunomodulation, electroactive regeneration, and staged therapeutic release. Additionally, the application of machine learning and multi-omics approaches provides predictive insights into how synthesis parameters, protein coronas, and cell responses may influence efficacy and toxicity, thus expediting the development of safe, biocompatible designs of silver nanocomposites. Realizing clinical potential requires reproducible fabrication, scalable manufacturing, and validation of dose parameters, oxidative stress responses, and immune compatibility. Clinically, silver nanocomposites have made significant progress, ranging from FDA-approved wound dressings and implant coatings to investigation platforms for cancer therapy, neural interfaces, and regenerative scaffolds. The future development of advanced silver nanocomposites will not be measured solely by their antimicrobial potency, but by integrating multiple orthogonal functions, including controlled ion release, mechanical reinforcement, electronic conductivity, and biological programming, within architectures verified through quantitative molecular readouts. Situated at the intersection of materials science, bioengineering, and regulatory science, silver nanocomposites offer a flexible platform poised to transform infection management, regenerative medicine, and precision therapeutics.

## Graphical abstract

**Figure:**
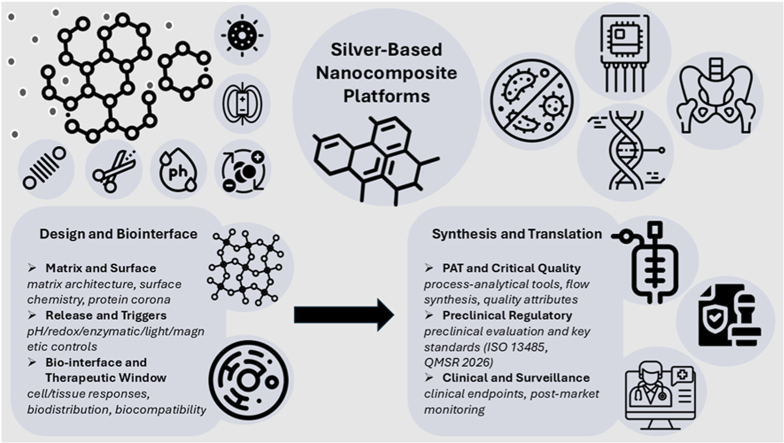

## 1 Introduction and scope

Silver has been used in medicine for thousands of years. Ancient Chinese, Mediterranean, Persian, and Phoenician societies stored food and water in silver containers to slow spoilage and reduce disease, an early example of surface-mediated antimicrobial control [1]. By the late nineteenth and early twentieth centuries, colloidal formulations of silver such as Collargol and Protargol were sold as antiseptics, and silver nitrate became a standard prophylactic agent against ophthalmia neonatorum via Credé’s method [2], one of the earliest population-scale infection-prevention policies [3]. In the United States, silver's modern status as a biocide was formalized in 1954 when the Environmental Protection Agency registered it under the Federal Insecticide, Fungicide, and Rodenticide Act [4,5].

The development of antibiotics has shifted attention away from metals; however, the rise of antimicrobial resistance (AMR) has brought silver back into the spotlight. In 2019, AMR was directly responsible for about 1.27 million deaths and contributed to almost 4.95 million deaths worldwide [6,7]. Projections warn that, without effective interventions, AMR could cause more than 39 million deaths by 2050, with nearly 2 million deaths each year by mid-century. Healthcare-associated infections (HAIs) compound this burden: roughly 3.2 million people in Europe acquire HAIs annually, leading to around 37,000 deaths, and in the United States, there are approximately 1.7 million HAIs and 99,000 associated deaths each year [8,9]. Drug-resistant Gram-negative infections alone cause an estimated 25,000 deaths annually in Europe. These statistics underscore the urgent need for new and alternative antimicrobial strategies [10,11]. Silver is particularly compelling in this context because it acts through multiple pathways, disrupting cell membranes, binding protein thiols, interfering with cellular respiration and DNA replication, and generating reactive oxygen species (ROS) that are distinct from most antibiotic targets [12]. When used with conventional drugs, it can resensitize resistant pathogens by lowering the minimum inhibitory concentration [[13], [14], [15], [16]].

What is fundamentally new today is scale. At dimensions below ∼100 nm, silver acquires tunable plasmonic and catalytic properties absent in the bulk metal, setting the stage for diverse new biomedical and technological applications [17,18]. These features enable silver optical readout, photothermal and photodynamic therapies, as well as catalysis-driven antimicrobial actions that are absent in the bulk material. Embedding silver nanoparticles (AgNPs) into engineered matrices further transforms their behavior, as polymers and hybrid hosts suppress agglomeration, modulate silver ion (Ag^+^) release, and decouple mechanical, optical, and biological properties. These capabilities are directly relevant to wound dressings, implant coatings, biosensors, and soft bioelectronics. Beyond biomedicine, AgNP networks can form conductive elements in flexible electronics, core–shell hybrids with semiconducting or magnetic layers that support imaging and targeted drug delivery, and mesoporous or yolk–shell structures that allow controlled ion release and optical tuning. Such versatility marks a shift from silver as a simple antimicrobial agent to silver as a programmable component [19,20], and the field is now moving toward sense–respond–heal platforms. Hierarchical designs assemble AgNPs into one-dimensional fibers or three-dimensional aerogels, combining high surface area with mechanical strength. Core–shell and yolk–shell structures layer silver with magnetic oxides, semiconductors, or porous shells, enabling magnetic guidance, light harvesting, controlled cargo release, and surface-enhanced Raman scattering (SERS) diagnostics. Stimuli-responsive matrices trigger the release of Ag^+^ and/or co-therapeutics in response to changes in pH, redox state, enzyme activity, or light. Biointeractive composites also incorporate extracellular-matrix components, cells, or extracellular vesicles (EVs) to pair antimicrobial action with immune modulation and tissue repair. These advances move the field beyond passive “kill” coatings toward adaptive materials tailored to biology [21,22].

Despite notable progress, research on silver nanocomposites still lacks comprehensive cross-disciplinary integration. Many studies focus solely on synthesis, polymer formulation, or surface chemistry, often overlooking the interconnected nature of mechanobiology, hierarchical architecture, responsive designs, and translational applications. This segmentation presents challenges, particularly given the urgent need to combat antimicrobial resistance (AMR) and HAIs, which necessitate holistic solutions [22]. To effectively connect structure–property–function relationships within practical clinical constraints, the field requires a unified scientific framework. Such a framework should correlate ion-release kinetics with tissue compatibility, localized surface plasmon resonance (LSPR) signatures with therapeutic windows, corona composition with cellular uptake and biodistribution, and manufacturing parameters with batch consistency. Recent evaluations highlight this need, advocating for the integration of advanced functionalization techniques with regulatory-compliant characterization methods and scalable manufacturing processes [23,24].

**Fig. 1:**
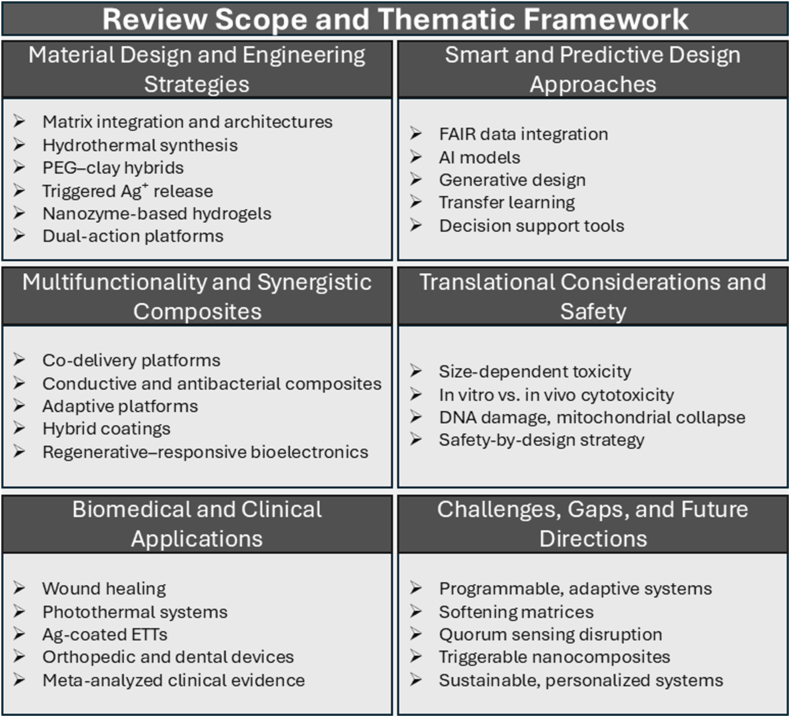
Schematic overview of the thematic structure and scope of this review. The organization reflects seven interlinked domains covered: Antimicrobial mechanisms of silver nanomaterials, material design and engineering strategies, multifunctional and synergistic composites, biomedical and clinical applications, smart and predictive design approaches, translational considerations and safety, and future challenges and directions. This framework integrates classical antimicrobial actions with next-generation innovations, emphasizing the transition from passive coatings to adaptive, programmable, sense–respond–heal platforms.

This review adopts a comprehensive and integrative approach, systematically examining the physicochemical properties of silver at the nanoscale, its antimicrobial mechanisms, and various biomedical applications. It evaluates the influence of different matrix modalities, including polymeric, carbon-based, ceramic, metallic, and porous structures, on nanoparticle dispersion, mechanical properties, optical responses, and ion transport. The discussion extends to stimuli-responsive platforms that enable controlled release of Ag^+^ and therapeutics, as well as biointeractive and living composites that facilitate antimicrobial activity alongside tissue regeneration. Additionally, it highlights the role of mechanobiology as a key design factor, linking material stiffness, topology, and porosity to biological outcomes. This review further explores multifunctional systems that integrate sensing, actuation, and therapeutic decision-making, alongside frameworks that leverage data-driven, multi-omics, and artificial intelligence (AI)-enabled methodologies for Safe-by-Design (SbD) development. The narrative shifts toward biomedical applications, addressing regulatory, safety, and manufacturing considerations, with an outlook on future opportunities. Finally, the review advocates for cross-disciplinary convergence among materials science, computation, and clinical practice. A schematic overview of the scope and organization of this review is provided in Fig. 1 to guide readers through the key conceptual and technical domains addressed.

Two principles underpin our approach. First, the value of silver in biomedicine is now measured not just by pathogen kill rates but by how effectively its effects can be quantified, modeled, and controlled. Techniques such as LSPR bands serve as real-time quality indicators; inductively coupled plasma mass spectrometry (ICP-MS) measures cellular uptake; protein corona composition predicts biodistribution; and digital twin simulations forecast variability to ensure reproducibility [23]. Second, successful translation relies equally on robust data standards and dose auditing alongside innovation. Interoperable descriptors, repositories that are findable, accessible, interoperable, and reusable (FAIR) [24,25], and regulator-ready analytics ensure that results are verifiable and applicable across laboratories. These are essential for achieving the clinical generalizability that stakeholders require [26,27].

**Fig. 2:**
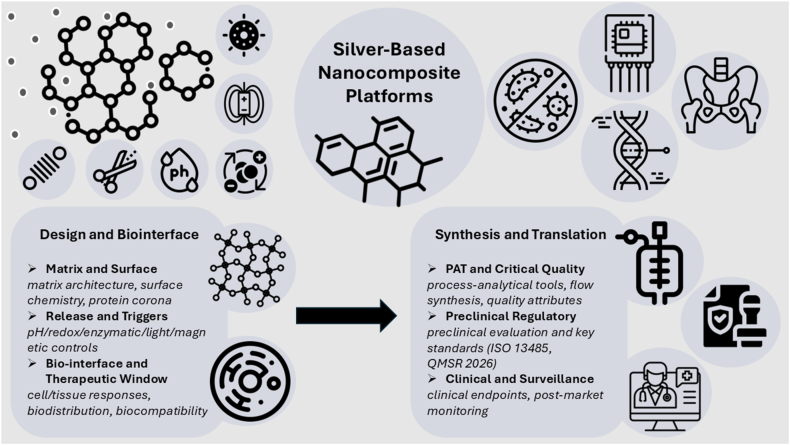
Design-to-translation map for silver nanocomposites. This schematic illustrates the integrated design-to-translation framework for silver nanocomposites in biomedicine. The top-left section shows how external triggers like pH, enzymes, light, and magnetism control silver ion release. This responsive flux enables antimicrobial, sensing, regenerative, and immunomodulatory functions, shown top-right. The bottom-left details matrix design and surface chemistry strategies that shape biological responses. The bottom-right outlines synthesis and translation pathways, including quality control, regulatory evaluation, and clinical endpoints. Together, the figure visualizes the “sense–respond–heal” platform evolution from material design to clinical readiness.

By bridging ancient practices with nanoscale engineering and uniting materials science, biology, computation, and regulation, we aim to provide a roadmap that is both ambitious and practical: ambitious in expanding the capabilities of silver nanocomposites and practical in demonstrating how they can be designed, benchmarked, and delivered safely at scale. An overview is provided in Fig. 2, which links the design architecture, stimulus-controlled silver ion dosing, and multifunctional biological outputs to regulatory-aligned translation pathways.

## 2 Fundamental properties and mechanisms of AgNPs

### 2.1 Unique properties and antimicrobial mechanisms

Silver at the nanoscale exhibits properties significantly more versatile than those of its bulk counterpart [28]. Due to their high surface area-to-volume ratios and quantum confinement of conduction electrons, the plasmonic, electrical, and catalytic behavior of AgNPs is highly sensitive to particle size and morphology [29]. In metallic nanoparticles, the collective oscillations of conduction electrons under electromagnetic excitation generate LSPR, which manifests as size- and shape-dependent optical absorption bands [30]. AgNPs are particularly notable for their narrow-band, high-intensity LSPR, which can be finely tuned by controlling particle geometry and the surrounding dielectric environment [[31], [32], [33]]. These plasmonic properties form the basis for a wide range of applications, including biosensing, molecular imaging, and photothermal therapy [34,35], where field enhancement and spectral tunability are critical [36].

Conjugation of AgNPs with photosensitizers such as porphyrins [37], phenothiazinium dyes [38], phthalocyanines [39], BODIPY derivatives [40], and ruthenium (II) complexes significantly enhance singlet oxygen (ˆ1O_2_) generation and phototoxic effects through LSPR, enabling synergistic photothermal–photodynamic therapy (PTT–PDT) for the eradication of pathogenic bacteria and tumor cells under visible or near-infrared (NIR) light. Beyond their therapeutic applications, AgNPs also serve as plasmonic enhancers in photocatalytic systems; for instance, conjugation with semiconductors such as TiO_2_ or WO_3_ extends light absorption into the visible range thereby enhancing photocatalytic performance. As such, AgNP-based plasmonic nanoconjugates represent a modular platform adaptable not only for theranostics [[41], [42], [43]], implantable devices, and clinically translatable biointerfaces, but also for photocatalysis-driven sensing and environmental remediation, all enabled by precise spatiotemporal activation [44].

Electrical conductivity is a hallmark of silver nanostructures and a result from the delocalization of conduction electrons across the surfaces of AgNPs. Well-dispersed AgNPs embedded in polymeric or carbonaceous matrices can form percolative networks, enabling efficient charge transport [45]. Recent advances have demonstrated that AgNP-based nanocomposites can be finely tailored to meet the demands of specific electronic and optoelectronic applications. For instance, AgNP-two-dimensional transition metal carbide/nitride (MXene) hybrids have been developed for ultrathin, stretchable strain sensors with high mechanical resilience and conductivity retention [46,47]. Inkjet-printed graphene–AgNP composites [48] serve as high-performance conductive inks for flexible radio-frequency identification tags and biomedical electronics [49]. Meanwhile, poly(3,4-ethylenedioxythiophene): polystyrene sulfonate (PEDOT:PSS)–AgNP films offer transparent conductivity and mechanical durability, outperforming conventional indium tin oxide in terms of stretchability and chemical robustness [50,51]. These examples underscore how the electrical properties of AgNPs can be strategically engineered and optimized for both single-function and multifunctional device platforms. Leveraging their conductive core for multifunctional integration, AgNPs incorporated into polymers such as polyethylene glycol (PEG) [52] and polyvinyl alcohol (PVA) [53] not only preserve electrical conductivity but can also be functionalized with magnetic components, such as Fe_3_O_4_ [54], to support magnetic resonance imaging, targeted drug delivery, and controlled release [55]. However, such modifications involving secondary coatings or dopants can affect optical transparency or charge mobility and require precise control over coating thickness and interfacial chemistry to preserve overall performance [56,57].

The high surface reactivity of AgNPs supports their catalytic function in redox reactions, including the reduction of organic dyes [58] and the generation of ROS [59,60]. Electron transfer processes, coupled with the continuous release of Ag^+^, contribute to their broad-spectrum antimicrobial activity. Released Ag^+^ ions bind to thiol-containing groups in bacterial proteins, disrupt membrane integrity, inhibit DNA replication, and induce intracellular oxidative stress [61,62]. The kinetics of Ag^+^ release are strongly dependent on particle size [63], shape, and surface chemistry [[64], [65], [66]]. Smaller AgNPs have been found to exhibit faster ion release due to their larger surface-area-to-volume ratio, which increases the proportion of reactive surface atoms available for oxidation and dissolution. Particle morphology also influences dissolution dynamics: high-energy crystal facets and anisotropic structures, such as plates or rods, tend to release Ag^+^ more rapidly than compact or spherical morphologies. Additionally, surface chemistry determines the rate and extent of ion liberation. Stabilizers and capping ligands such as citrate can form passivation layers that slow oxidation, whereas uncoated or weakly bound surfaces promote faster and less controlled release. Composite systems can modulate ion diffusion, influencing both antimicrobial efficacy and cytotoxicity. Studies have demonstrated that graphene oxide scaffolds, PVA-based hydrogels, chitosan–PVA films, and PEG–polyvinylpyrrolidone (PVP)–chitosan polymer blends are among the most extensively studied AgNP nanocomposites capable of regulating Ag^+^ diffusion through tunable release profiles, thereby reducing burst toxicity and ion flux while preserving antibacterial performance [[67], [68], [69]]. Crosslinking agents, such as glutaraldehyde, citric acid, and sodium tripolyphosphate, further enhance matrix integrity and control porosity, enabling sustained Ag^+^ release. Surface functionalization with PVP, PEG, or plant-derived polyphenols can also enhance colloidal stability, inhibit nanoparticle aggregation, and regulate interactions with biological systems [[70], [71], [72], [73]].

### 2.2 Hybrid and core–shell nanostructures

**Fig. 3:**
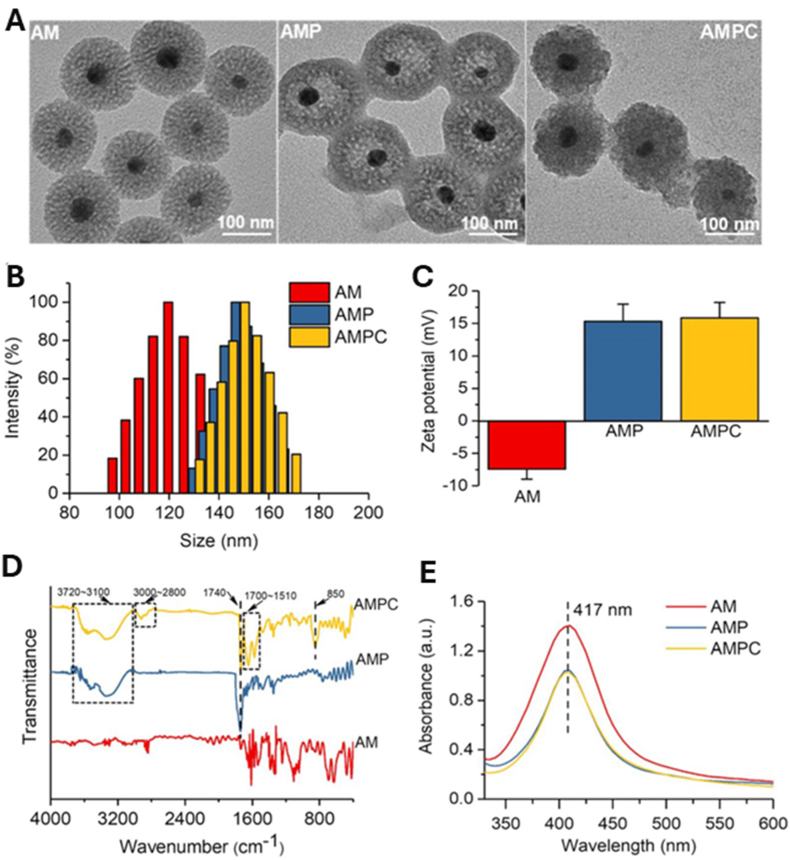

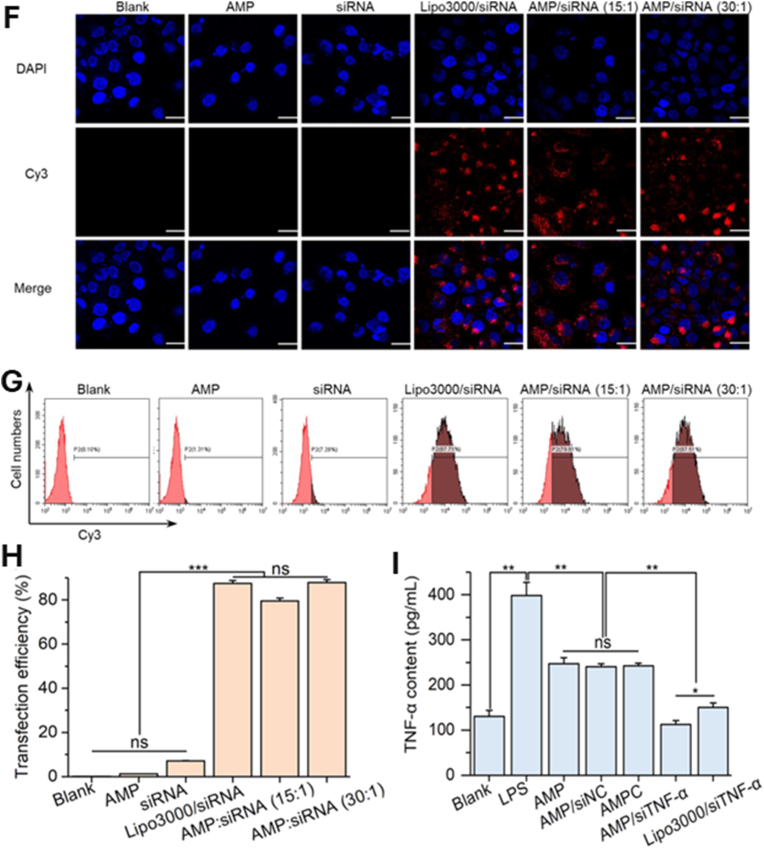

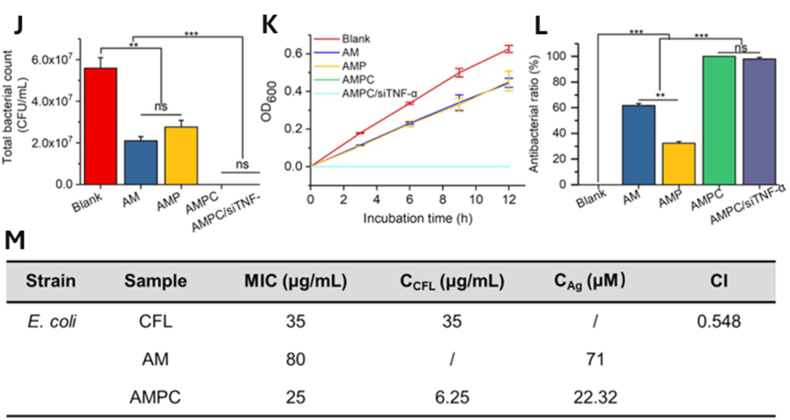

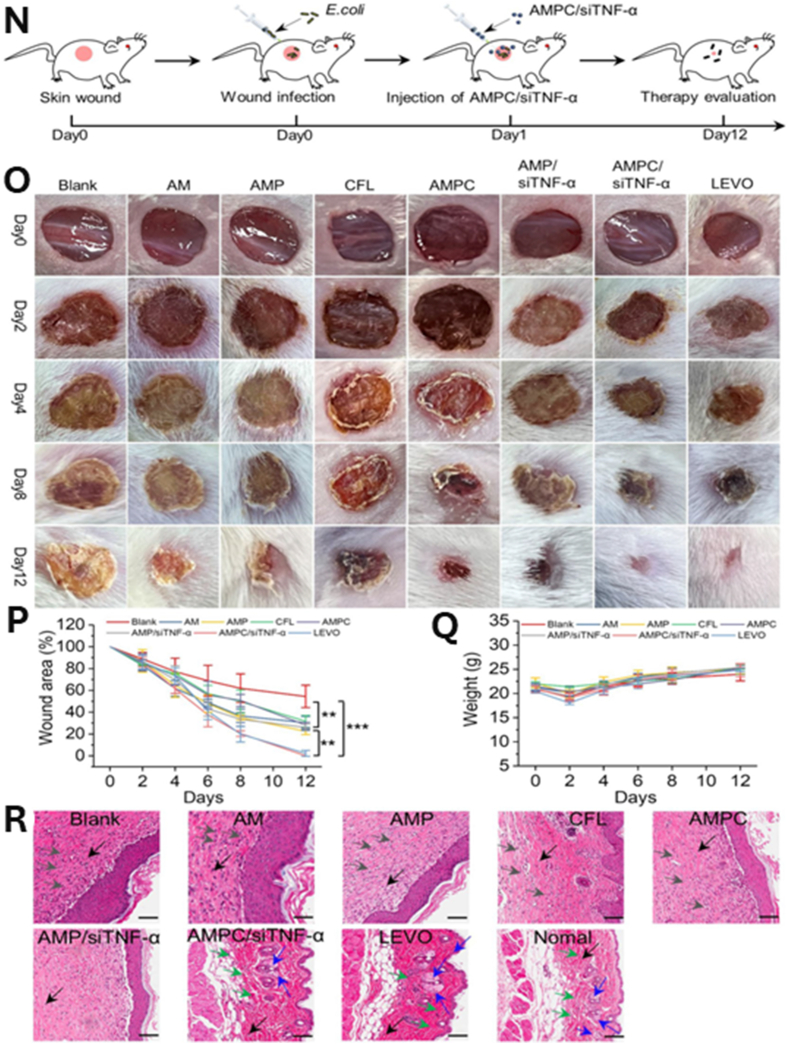
(A–E) Characterization of AMPC (Ag@MSNs/PEI-PEG-CD): (A) Transmission electron microscopy reveals the morphology of AM (Ag@MSNs), AMP (Ag@MSNs/PEI-PEG), and AMPC. (B) Dynamic light scattering analysis shows size distribution, and (C) zeta potential measurement confirms successful surface modification. (D) Fourier transform infrared and (E) ultraviolet–visible absorption spectra validate the chemical structure and optical properties. (F–I) siRNA delivery and gene silencing in lipopolysaccharide-induced RAW264.7 cells: (F) Fluorescence microscopy images show intracellular siRNA delivery using Cy3-labeled siRNA and DAPI nuclear staining. (G) Flow cytometry histograms and (H) quantitative analysis demonstrate high transfection efficiency of AMPC compared to controls. (I) Tumor necrosis factor-alpha (TNF-α) protein expression levels confirm effective gene silencing by AMPC/siTNF-α. (J–M) Antibacterial performance against *Escherichia coli* (*E. coli*): (J) Bacterial colony-forming unit (CFU) counts, (K) growth curves, and (L) antibacterial ratios illustrate enhanced antibacterial activity of AMPC/siTNF-α. (M) Minimum inhibitory concentration (MIC) values, concentration of cefalexin (C_CFL_), and concentration of silver (C_Ag_) indicate synergistic antibacterial efficacy, with combination index (CI) values confirming additive or synergistic effects. (N–R) *In vivo* wound healing efficacy in *E. coli*-infected skin models: (N) Schematic shows treatment procedure, including infection and therapeutic intervention. (O) Photographic tracking of wound closure across treatment groups from day 0 to day 12. (P) Quantitative analysis of wound area reduction and (Q) weight tracking demonstrate safety and effectiveness. (R) Histological images (hematoxylin and eosin staining) of skin tissue reveal tissue regeneration, inflammatory response, and structural integrity in treated groups. Abbreviations: AM: Ag@mesoporous silica nanoparticles (MSNs); AMP: Ag@MSNs coated with polyethyleneimine-polyethylene glycol (PEI-PEG); AMPC: Ag@MSNs/PEI-PEG-cyclodextrin (CD); siRNA: small interfering RNA; CFL: cefalexin; LEVO: levofloxacin; Cy3: cyanine-3 fluorescent dye; DAPI: 4′,6-diamidino-2-phenylindole. Adapted from Liu et al., 2022, J Nanobiotechnol [86], under CC BY 4.0, https://creativecommons.org/licenses/by/4.0; labels were edited.

Core–shell nanostructures represent a rational platform for engineering the spatial distribution of functional domains, enabling potential synergistic coupling between the inner core and outer shell [74,75]. In these architectures, silver may serve either as an active core encapsulated within stabilizing or stimuli-responsive matrices or as a surface-deposited shell that imparts plasmonic, antimicrobial, or catalytic functionalities [76]. Silver-core architectures such as Ag@SiO_2_ [77], Ag@ zeolitic imidazolate framework ZIF-8 [78], Ag@poly(lactic-co-glycolic acid) (PLGA) [79], and Ag@poly(N-isopropylacrylamide) [80] rely on encapsulation-driven stability and release modulation, where silver is physically confined within polymeric or inorganic shells to achieve site-specific Ag^+^ release under physiologically relevant conditions [81]. This makes them ideal for drug delivery, as the shell can act as a drug-loading matrix, regulating release kinetics, preserving payload bioactivity, and minimizing systemic toxicity. They support localized therapy and multifunctional release platforms in wound healing, infection control, and implant coatings [[82], [83], [84]]. An illustrative example of such multifunctionality is presented in Fig. 3, which highlights an antimicrobial peptide-functionalized AgNP–mesoporous silica nanoplatform designed for the co-delivery of ciprofloxacin and siRNA. The integrated system demonstrates simultaneous antimicrobial action, siRNA-mediated cytokine silencing, and regenerative support in a wound infection model. Structural characterization confirms colloidal stability and drug incorporation, while biological assays reveal enhanced cellular uptake, efficient gene silencing, suppressed bacterial growth, and accelerated wound closure. This example exemplifies how architecture-guided design can yield sense–respond–heal capabilities, supporting the broader utility of core–shell silver nanostructures in advanced biomedical applications [82]. Silver-shell nanocomposites such as Fe_3_O_4_@Ag [85], TiO_2_@Ag [86], and ZnO@Ag [87], on the other hand, leverage interfacial synergy between core and shell to achieve architecture-mediated performance enhancement. In these systems, functional cores, typically magnetic or photocatalytic, are coated with silver shells anchored onto their surfaces, maximizing AgNP surface availability for direct biointerface interaction [88]. These systems are well-suited for surface-disruptive therapies, where plasmonic heating, ROS generation, or magnetic targeting play a central role [89,90]. The silver shell also promotes membrane disruption and enhances permeability, which is critical in photothermal ablation, biosensing, SERS-based diagnostics, and synergistic therapy [91,92].

### 2.3 Hierarchical and mesostructured architectures

Hierarchical and mesostructured nanomaterials expand the design space beyond classical core–shell architectures by incorporating multiscale geometric complexity, enabling improved surface functionality, mass transport, and light–matter interactions. These constructs often feature branched, porous, or multilayered interfaces, where structural anisotropy and internal compartmentalization facilitate performance enhancements in biosensing, catalysis, and antimicrobial platforms [93]. As a model system, ZnO–CuO/Ag nanowire arrays demonstrate the concept, where thermally grown ZnO nanowires are sequentially coated with CuO and decorated with AgNPs via vacuum thermal evaporation [93]. This multicomponent structure forms a hierarchically textured surface that exhibits low water adhesion and self-cleaning behavior, alongside potent antibacterial activity due to synergistic interactions among the metal oxides and surface-bound silver [92]. The dry-process synthesis is scalable and compatible with flexible substrates, making it a viable approach for large-area antimicrobial coatings [94].

For additional functional tunability, mesoporous and yolk–shell constructs are developed to control void space and shell permeability [95,96]. For instance, Ag@WO_3_ core–shell nanospheres [97] fabricated via pulsed laser ablation, show enhanced photodetector performance, leveraging plasmon-mediated charge separation and internal scattering within the mesoporous shell [98]. AgNP@ metal–organic framework (MOF) [99] yolk–shell systems (e.g., Ag@ZIF-8 yolk–shells) [100] further integrate crystalline porous shells with AgNP cores, allowing for size-selective molecular gating, controlled ion exchange, and chemical stimulus responsiveness [[101], [102], [103], [104]]. These features enable precise control over antimicrobial ion release and are increasingly explored in smart wound dressings, biosensors, and ion-selective membranes. In these systems, shell thickness and porosity are engineered to modulate optical properties and ion diffusion, both critical in optoelectronic and catalytic applications. Ag@TiO_2_ hollow spheres [105] represent another class of hierarchical structures, where light-responsive TiO_2_ shells encapsulate plasmonic AgNPs. These systems exhibit efficient photocatalytic performance due to enhanced light harvesting and ROS generation, relevant for surface disinfection and pollutant degradation [106].

### 2.4 Property–function relationships and design considerations

The functional performance of silver-based nanomaterials emerges from a convergence of architectural design, interfacial chemistry, and compositional tuning (Fig. 3). While size, shape, and silver loading influence plasmonic behavior and ion release, these parameters alone rarely determine application-relevant outcomes. Instead, functionality is more accurately understood as a consequence of how structural and chemical features are integrated across scales.

Key design considerations include the trade-off between ion release rate and cytotoxicity, the need to balance surface accessibility with stability, and the role of shell composition in modulating interactions with biological or catalytic environments. For example, porous shells enhance diffusion and sensing but may compromise mechanical durability unless reinforced by hybrid matrices. Maximizing antimicrobial efficacy may require high silver surface exposure, whereas minimizing oxidative stress in mammalian cells demands more controlled release kinetics. Another critical aspect is the choice of interfacial modifiers, such as organic linkers, biopolymers, or oxide coatings, which can significantly influence colloidal stability, conjugation potential, and charge transport. In multifunctional systems, the spatial arrangement of silver relative to other active domains, such as magnetic, fluorescent, and enzymatic, must be precisely engineered to preserve or enhance each functional modality with little interference.

**Fig. 4:**
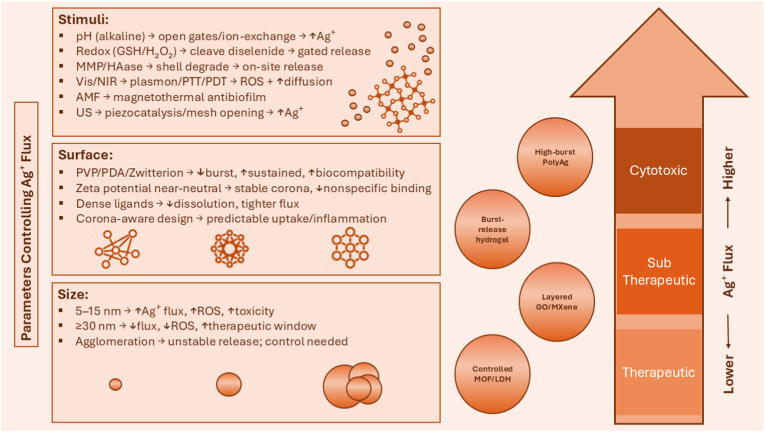
Parameters governing Ag^+^ flux and the therapeutic window in silver nanocomposites. The organization Ag^+^ transport is regulated by a combination of stimuli-responsive triggers, surface chemistry, particle size, and matrix architecture. These parameters collectively define whether silver release falls within the therapeutic window or drifts toward sub-therapeutic or cytotoxic zones. Internal (e.g., pH, redox, enzymatic) and external (e.g., light, AMF, ultrasound) stimuli modulate release via structural or catalytic changes. Surface coatings such as PVP, PDA, and zwitterions tune burst *vs.* sustained release and modulate protein corona effects. Size-dependent dissolution, aggregation, and matrix design (e.g., hydrogels *vs.* MOFs) further influence release kinetics. Together, these factors enable programmable and context-specific silver delivery.

Rather than treating these features as independent design variables, successful nanocomposite development requires a systems-level approach, where structural hierarchy, chemical functionality, and performance outputs are co-optimized. Fig. 4 illustrates how these interdependent variables, including matrix chemistry, particle size, aggregation state, and surface modification, jointly modulate Ag^+^ flux. This flux defines a therapeutic window bounded by sub-therapeutic activity on one end and cytotoxicity on the other. For example, small particles (<15 nm) with unstable matrices may result in excessive Ag^+^ release and toxicity, whereas well-engineered hybrid systems, such as MOF-coated composites, offer controlled flux and improved biocompatibility. Designing silver nanocomposites within the therapeutic window is crucial for achieving efficacy without adverse biological outcomes. This design logic provides the foundation for the following sections, which explore how silver nanostructures are embedded within diverse matrices and programmed to respond to environmental or biological cues.

## 3 Matrix integration and advanced architectures

### 3.1 Polymeric nanocomposites and hierarchical scaffolds

**Fig. 5:**
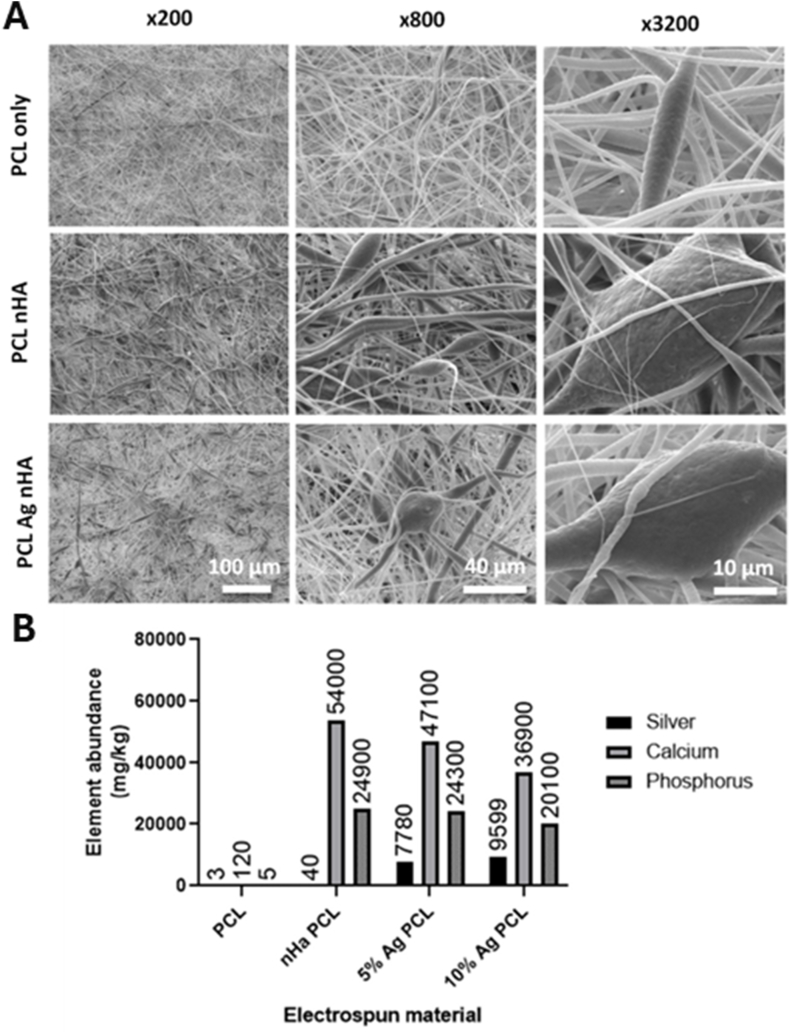

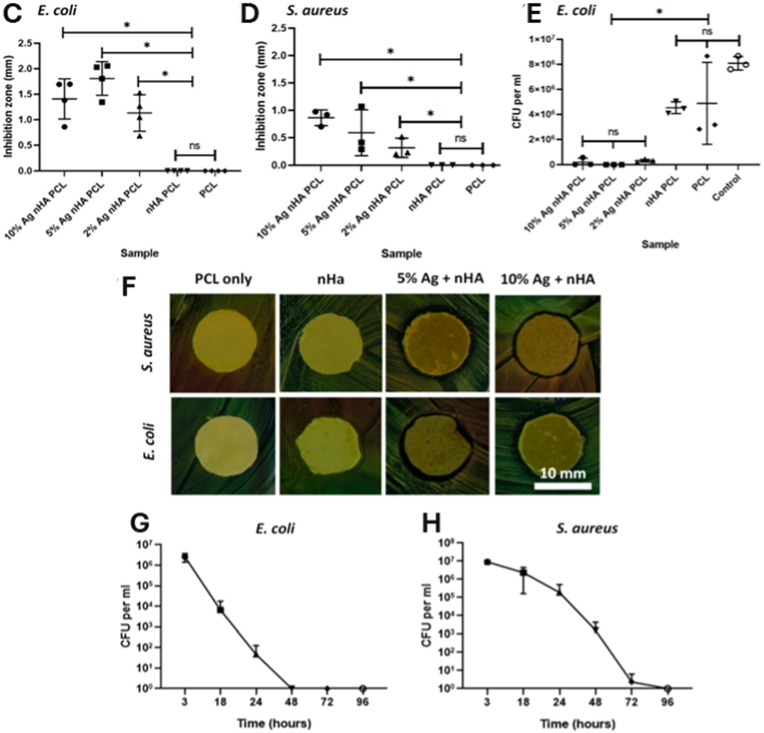
(A, B) Scaffold fibers containing nHA: (A) SEM micrographs of electrospun scaffold. Top: Polycaprolactone (PCL) scaffold. Middle: PCL scaffold containing nHA. Bottom: PCL scaffold containing 10 mol.% Ag nHA. (B) Inductively coupled plasma (ICP) analysis. (C–H) Antibacterial studies on *E. coli* and *S. aureus* bacteria using the electrospun scaffold: Agar diffusion test against (C) *E. coli* and (D) *S. aureus*. (E) Disks of electrospun samples suspended in phosphate-buffered saline (PBS) containing bacteria, measuring viable colonies after 24 h. (F) Optical images of the area of inhibition surrounding a 12-mm disk of scaffold containing either 10 mol.% Ag or plain PCL and tested against *S. aureus* and *E. coli*. (G) Bacteria count after *E. coli* are suspended in PBS and exposed to a disk of electrospun 10 mol.% Ag nHA for 3, 18, 24, 48, 72, and 96 h. (H) Same as (G) but using *S. aureus*. (I–L) Cell toxicity from the electrospun scaffold with different concentrations of silver nHA assessed by cell metabolic activity via PrestoBlueTM assay: A 48 h noncontact toxicity test in the media above a monolayer of (I) 3T3 cells and (J) primary rat MSCs. (K) Cell metabolism after 21 days of direct culture with the cells seeded on top of the scaffold using metabolic activity at each time point. (L) Live/dead staining of MSCs cultured on electrospun scaffolds after 21 days. Abbreviations: nHA: nanohydroxyapatite; MSC: mesenchymal stem cell; a.u.: arbitrary units. Adapted from Paterson et al., 2020, J Funct Biomater [132], under CC BY 4.0, https://creativecommons.org/licenses/by/4.0; labels were edited.

Polymers as highly adaptable hosts for AgNPs enable the design of multifunctional materials that integrate mechanical durability, structural tunability, and controlled antimicrobial activity [107]. Recent advances in AgNP–polymer integration have shifted from simple physical blends to engineered matrices, such as electrospun nanofibers [108], hybrid hierarchical scaffolds [109], and biopolymer-based hydrogels [110] that support precise nanoparticle dispersion, sustained ion release, and therapeutic functions [111,112]. Electrospun chitosan/polyethylene oxide (PEO) fibers [113], for instance, exhibit tensile strengths comparable to those of human skin and inhibit the growth of both *Pseudomonas aeruginosa* (*P. aeruginosa*) and *Staphylococcus aureus* (*S. aureus*). When co-loaded with agents like curcumin [114], sulfanilamide [115], or chondroitin-4-sulfate [116], these fibers also support anti-inflammatory or regenerative outcomes without compromising mechanical strength [[117], [118], [119], [120]]. Nanostructural features, such as fiber diameters and porosity, facilitate gas and nutrient exchange, promoting cellular adhesion [121,122]. To achieve a precise spatial distribution of silver within biomedical matrices, hybrid electrospinning–electrospraying techniques have been developed to embed AgNP-loaded PVA microspheres within PCL scaffolds, yielding hierarchical scaffolds for localized therapy [123]. An illustrative example is shown in Fig. 5, which presents an electrospun PCL scaffold incorporating silver-doped nanohydroxyapatite (Ag-nHA) for orthopedic and dental bone applications. The figure highlights key performance features, including antimicrobial efficacy against both *Escherichia coli* (*E. coli*) and *S. aureus*, as well as long-term cytocompatibility with fibroblasts and mesenchymal stem cells.

Biopolymer-derived hydrogels based on galactomannan or κ-carrageenan serve dual roles as reducing agents and structural matrices, with their concentration influencing the size of AgNPs, swelling behavior, and release kinetics [[124], [125], [126]]. Additional strategies, such as gamma irradiation [127,128] of polyacrylamide–chitosan networks, enable simultaneous crosslinking and *in situ* Ag^+^ reduction, resulting in antifungal or anticancer hydrogels with defined mechanical properties. Hydrothermal synthesis methods further eliminate the need for surfactants, generating monodispersed AgNPs in fully aqueous systems with optimized biocompatibility [129,130]. Regardless of the platform, antimicrobial performance is not determined solely by the presence of AgNPs, but by their integration into the polymer network, which is influenced by matrix composition, interfacial interactions, and processing conditions. For instance, organophilic clays dispersed within PEG improve AgNP dispersion and reinforce tensile properties [131,132]. Plant-extract-mediated synthesis within biodegradable poly lactic acid (PLA)–PEG or chitosan matrices leverages green chemistry to achieve both biostability and structural control [133,134]. Various polymeric nanocomposites demonstrate that the strategic design of fiber morphology, hydrogel porosity, and nanoparticle positioning enables the co-optimization of mechanical performance, Ag^+^ release, and secondary therapeutic functions, making them highly effective platforms for localized antimicrobial delivery, wound healing scaffolds, and infection-resistant biomedical coatings.

### 3.2 Carbon-based hosts

Carbon-based materials ranging from two-dimensional graphene oxide (GO) and reduced graphene oxide (rGO) to one-dimensional carbon nanotubes (CNTs) [135] and zero-dimensional carbon quantum dots (CQDs) provide structurally diverse and functionally tunable frameworks for AgNP integration. These carbonaceous substrates not only enhance electrical conductivity and mechanical reinforcement but also facilitate the design of nanocomposites with broad-spectrum antimicrobial, anticancer, sensing, and catalytic functionalities [136,137].

Among carbon-based hosts, GO is particularly notable for its surface chemistry. The oxygen-containing functional groups of GO promote uniform nucleation and dispersion of AgNPs in aqueous environments [138]. GO–AgNP composites, particularly those synthesized via green methods using *Calendula officinalis*, have demonstrated complete inhibition of *E. coli* and *methicillin-resistant Staphylococcus aureus* (MRSA) at optimal silver loadings [139,140]. However, excessive Ag concentrations lead to nanoparticle aggregation and a reduction in antimicrobial efficacy. Compared to GO, rGO exhibits higher electrical conductivity and a partially restored sp^2^-carbon network. rGO–AgNP hybrids outperform both standalone treatments with rGO or AgNPs in suppressing breast cancer [141] mammosphere formation and downregulating pluripotency-associated genes such as homeobox protein NANOG and octamer-binding transcription factor 4 (OCT4) in MCF-7 cells [142]. *In vivo*, biogenic rGO–Ag nanocomposites have been shown to reduce tumor burden in murine ascites carcinoma models, highlighting their potential in localized cancer therapy [143].

While CNTs, as one-dimensional systems, have also been explored for applications such as photothermal therapy, imaging, and targeted drug delivery in cancer studies, they have been more commonly featured in materials science and device-oriented research. CNTs, particularly multi-walled CNTs (MWCNTs), contribute high aspect ratios and robust mechanical properties. Functionalized MWCNTs provide anchoring sites for AgNPs, forming conductive nanonetworks suitable for electronic, antimicrobial, and thermal management applications [144]. Solvent-free green syntheses, such as gallic acid-mediated reduction [145] or tin(II) ion displacement reactions, produce CNT–Ag nanohybrids with significantly reduced resistivity, enhanced thermal conductivity, and improved shear strength when integrated into flexible pastes and coatings [146]. These properties make CNT–Ag systems attractive for electromagnetic interference shielding and wearable electronics [147,148].

Moving toward zero-dimensional carbon platforms, CQDs represent a rapidly evolving class of carbon nanomaterials with intrinsic fluorescence, high surface-to-volume ratios, and multifunctional potential. Doped CQDs decorated with AgNPs, such as sulfur- and nitrogen-co-doped variants, have demonstrated potent antimicrobial activity against multiple pathogens, including MRSA and *Candida albicans*, while maintaining low cytotoxicity [149]. In environmental applications, green-synthesized CQD–Ag hybrids have achieved greater than 99 % catalytic degradation of methylene blue and methyl orange under ambient conditions [150]. Ag–CQD conjugates immobilized on paper-based platforms or functionalized with small-molecule ligands (e.g., metformin-binding moieties) have enabled ultrasensitive detection of biomolecules via fluorescence quenching, with detection limits in the nanomolar range, supporting their use in next-generation point-of-care diagnostics [[151], [152], [153], [154]].

In addition to the commonly studied AgNP–carbon nanocomposites discussed above, alternative platforms such as activated carbon [155], carbon black [156], and diamond-like carbon [157] have been explored for silver integration. Although less frequently investigated, these systems exhibit antimicrobial and structural benefits and may gain further relevance with continued advances in surface functionalization and biomedical optimization.

### 3.3 Ceramic and glassy matrices

Ceramic and glassy matrices offer a robust platform for AgNP integration due to their inherent thermal stability, chemical inertness, and compatibility with high-temperature processing. These inorganic hosts not only withstand aggressive environments but also provide rigid, immobilizing frameworks that support long-term antimicrobial performance [158,159]. A well-studied example is the co-sputtering of zirconia and silver onto nanofibrous membranes, which yields amorphous ZrO_2_ matrices [160] encapsulating silver nanoclusters. These coatings significantly enhance membrane hydrophobicity and exhibit potent antimicrobial effects, resulting in over 99 % reduction of *Staphylococcus epidermidis*, Bacillus subtilis (*B. subtilis*), and *Listeria monocytogenes* during filtration [[161], [162], [163]]. Their functionally textured surfaces facilitate bacterial repulsion while maintaining structural integrity, an outcome further replicated in silver-doped zirconia capillary membranes, which deliver an 8-log reduction in bacterial growth with minimal silver leaching over multiple filtration cycles [[164], [165], [166]]. In titania-based systems, where polydopamine (PDA) is employed to reduce and immobilize Ag^+^ onto TiO_2_ nanoparticles, stable silver confinement is also achieved. These TiO_2_–PDA–Ag nanohybrids, embedded in silicone matrices, preserve the silicone's mechanical properties and suppress *B. subtilis* by 99.9 % and completely inhibit *E. coli* adhesion [[167], [168], [169]]. Another example of rigid inorganic scaffolds with organic modifiers is polydopamine/tannic acid coatings on titanium, which tether AgNPs to oxide surfaces while maintaining fibroblast viability and promoting hydrophilicity, thereby supporting their clinical utility as biocompatible antibacterial coatings [[170], [171], [172]]. While all systems rely on amorphous or nanocrystalline ceramic scaffolds, they diverge in how silver is integrated: through glass-phase encapsulation [173], surface-bound chemical reduction [174,175], or deposition into electrochemically formed nanotubes [176,177]. In zirconium–silver alloy-derived anodized nanotubes [178], controlled release from high-aspect-ratio nanostructures has enabled ≥99.9 % killing of *S. aureus* and over 99 % reduction of *E. coli*. Some systems emphasize durable surface adhesion and anti-fouling functionality, while others prioritize tunable Ag^+^ release for extended biocidal action, depending on how silver is incorporated into the ceramic host as a highly versatile, structurally rigid, and chemically adaptive platform for multifunctional AgNP coatings used in water treatment, implant protection, and anti-biofilm technologies.

### 3.4 Metallic matrices and magnetic hybrids

Embedding AgNPs into metallic matrices yields durable, wear-resistant coatings with intrinsic antimicrobial activity. Doping intermetallic alloys such as Ti_3_Au with trace silver is a notable example; mosaic sputtering of just 0.2 atomic percent Ag raises hardness to 14.7 GPa, a 360 % improvement over conventional Ti_6_Al_4_V, while preserving cytocompatibility. These coatings also eradicate bacterial pathogens within 20 min, making them promising for orthopedic and dental implants [[179], [180], [181], [182]].

Composites such as Fe_3_O_4_@SiO_2_@PDA@Ag [183] and NiFe_2_O_4_@Ag [184] are evaluated for their superparamagnetic and magnetically responsive properties, enabling field-guided localization, magnetic hyperthermia, and magnetically assisted retrieval. In practice, silver-conjugated superparamagnetic iron oxides eradicate MRSA biofilms more effectively under an external field [185,186]. Mesoporous hollow Fe_3_O_4_ achieves similar effects through magnetic hyperthermia [187]. Magnetite–silver hybrids also deliver higher bacterial killing under alternating magnetic field (AMF) than at equivalent isothermal temperatures, pointing to field-mediated mechanisms at the microbe material interface. Magnetically recyclable Ag–oxide composites offer additional functionality, enabling capture and reuse, thereby limiting Ag^+^ release [[188], [189], [190], [191]]. CoFe_2_O_4_@Ag core shells and Ag-decorated CoFe_2_O_4_ on two-dimensional (2D) supports, both cobalt ferrite–silver hybrids, share strong field-addressable antibiofilm activity while retaining superparamagnetism, making them suitable for magnetically guided antimicrobial strategies [[192], [193], [194], [195], [196], [197]]. MnFe_2_O_4_–Ag hybrids add dual-mode hyperthermia capability (magnetothermal + photothermal), extending potential applications to tumor as well as biofilm treatment [[198], [199], [200]]. Studies focused on implant coatings have shown that TiN–Ag multilayers and Ag-bearing high-entropy alloy [201,202] coatings (laser-cladded FeCrNiAlTiAg_x_) represent metallic matrix systems that combine hardness, wear resistance, corrosion protection, and antibacterial performance [[203], [204], [205]]. Multilayer [YSZ/HAp–Ag]_n_ stacks align with this implant-focused approach, integrating osteoconductive interfaces with Ag-based bactericidal function while maintaining adhesion and mechanical strength [[206], [207], [208], [209]].

### 3.5 Metal–organic frameworks and porous hosts

Porous structures such as metal–organic frameworks (MOFs) and aerogels offer high surface area, uniform binding sites, and tunable pores that stabilize ultrasmall silver and control mass transport [[210], [211], [212], [213]]. Silver anchored on UiO-66(Zr) derivatives, for instance, form heterostructures with capabilities in gas sensing, photocatalysis, antimicrobial delivery, and drug transport; Ag_2_O-decorated UiO-66(Zr) achieves hydrogen sulfide detection limits of 1 part per million (ppm) at room temperature due to the strong H_2_S adsorption affinity of silver oxide [214]. Silver-based MOF nanostructures have been investigated for their antibacterial activity, with numerous studies demonstrating their ability to inhibit or kill a broad range of bacterial strains. UiO-66, ZIF-8–Ag core–shells modulate Ag^+^ release and co-load antibiotics for sustained antimicrobial action with reduced burst, showing inhibition of Gram-negative bacteria such as *E. coli in vitro* [215]. MIL-101(Fe)–Ag and related Fe-MOFs leverage redox-active nodes to enhance ROS generation, disrupting *P. aeruginosa* biofilms in both static and flow cell models [216]. HKUST-1(Cu)–Ag yields SERS-active antimicrobial membranes effective against Gram-positive species, including *S. aureus*, while enabling simultaneous pathogen detection via SERS [217].

MOF-derived carbons and oxides preserve hierarchical porosity for immobilizing silver in reusable catalytic, disinfection, and water purification platforms, with examples such as ZIF-67-derived cobalt–carbon–Ag hybrids and MIL-53 (Al)-derived porous carbons functionalized with Ag for dye degradation and microbial inactivation in wastewater [218,219]. Cellulose or graphene-based aerogels decorated with silver, including silver–nanocellulose aerogels and Ag-graphene oxide aerogels, deliver lightweight, compressible filters that achieve high bactericidal rates with low Ag leaching. Across these systems, the porous framework concentrates reactants, stabilizes the metal phase, and enables controlled release or selective uptake, producing multifunctional materials suited to environmental remediation, sensing technologies, and advanced antimicrobial therapies [220].

### 3.6 Core–shell, mesoporous, and layered architectures

These architectures are designed to deliver controlled Ag^+^ release, provide antimicrobial activity under ambient visible light, protect against corrosion, and allow for facile recovery and reuse. In mesoporous silicas such as SBA-15 and MCM-41, the uniform arrangement of pores, typically 5–10 nm in diameter with defined connectivity and tortuosity, regulates Ag^+^ diffusion. This structural precision slows ion transport, suppressing the burst release seen in less organized hosts and ensuring a more predictable antimicrobial effect. Layered double hydroxides such as Zn–Al LDH [221] operate through an interlayer-mediated ion-exchange mechanism: the galleries act as ion reservoirs for sustained release, while the layered structure forms a durable, corrosion-resistant coating on metal surfaces, maintaining stability through repeated wet–dry and saline cycles [[222], [223], [224], [225], [226], [227]].

Many of these systems incorporate visible-light responsiveness through engineered semiconductor junctions, enabling photochemically driven antimicrobial and pollutant degradation pathways. Examples include g-C_3_N_4_–Ag and ternary Ag_2_O/TiO_2_/g-C_3_N_4_, which align their electronic bands to promote charge separation under low-intensity light (∼100–500 lx). This alignment drives the formation of ROS (•OH, O_2_•^-^, ^1^O_2_), which inactivates microbes within minutes and achieves greater than 90 % degradation of dyes and antibiotics in laboratory tests [228,229]. Few-layer WS_2_ decorated with silver functions as a visible-light-active 2D semiconductor for thin bactericidal coatings and label-free sensing. Plasmonic Ag@AgCl absorbs indoor light to enhance interfacial charge separation, and when paired with a ferrite (Fe_2_O_3_) core, gains magnetic properties for easy capture and reuse without loss of photocatalytic efficiency over multiple cycles [230,231].

These structures are also effective in water-pollution control, where precise flow pathways and localized antimicrobial activity are critical. Incorporating silver into lamellar GO or MXene membranes creates aligned nanochannels that direct fluid flow, maintaining high water flux (>1000 L m^−2^ h^−1^) and achieving over 99 % oil rejection. Silver positioned along the channel walls provides illumination-independent, localized antimicrobial and anti-fouling activity, and confinement within the lamellae helps prevent silver leaching while preserving separation performance during repeated use [[232], [233], [234]].

### 3.7 Summary

Matrix integration transforms AgNPs from simple colloidal systems into multifunctional materials relevant to nanomedicine, energy conversion, catalysis, environmental remediation, and other advanced applications. Each host category, polymers, carbon allotropes, ceramics, metals, and porous frameworks, confers distinct mechanical, chemical, and mass-transport properties, while advanced architectures such as core–shell, mesoporous, and hierarchical scaffolds leverage spatial organization to achieve synergistic performance. Structure–property–function relationships identified in recent nanomaterials research highlight the critical importance of controlling particle size, size distribution, and interfacial chemistry. These matrix platforms define not only the static structural and mechanical behavior of silver nanocomposites but also their dynamic responsiveness to environmental cues. The same chemical interactions, porosity, and interfacial chemistries that govern baseline Ag^+^ release also dictate how these systems respond to physiological or externally applied stimuli such as pH, redox potential, light, or magnetic fields. Accordingly, the next section expands on how these matrix architectures are harnessed to achieve controlled, stimulus-triggered functions, linking material design directly to adaptive performance. Key integration strategies and biomedical implications of major matrix classes are summarized in Table 1.

**Table 1:** Matrix-driven design of silver nanocomposites: Classes, integration strategies, and functional outcomes. This table displays a range of host matrices, from biopolymers to metals and porous frameworks, explaining how each type affects the incorporation of AgNPs, the resulting physicochemical and biological properties, and the associated challenges. It functions as a comprehensive guide for designing Ag nanocomposites for specific biomedical applications.

Matrix class	Representative integration method	Tailored physicochemical advantage	Primary biomedical function	Challenge or limitation	Representative application	Reference
**Natural biopolymer** (chitosan, alginate, bacterial cellulose, silk fibroin, konjac glucomannan)	*In-situ* reduction or electrospinning enabling AgNP immobilization within hydrogel or film networks	High elasticity, hydrophilicity, and uniform AgNP dispersion for controlled Ag^+^ diffusion	Moist-wound healing Promotion and antimicrobial protection	Enzymatic degradation and low mechanical resilience under physiological load	Bacterial-cellulose–Ag membranes, chitosan–Ag hydrogels, alginate–Ag scaffolds for tissue repair	[295,463]
**Synthetic polymer** (PCL, PEG, PVA)	*In-situ* polymerization or reduction distributing AgNPs within polymer chains	Enhanced mechanical robustness and tunable Ag^+^-release kinetics	Provides durable antimicrobial barrier and implant compatibility	Limited biodegradability and residual toxicity	PCL–Ag nanofiber coatings and PEG–Ag films for implants	[464,465]
**Hybrid bio-organic matrix** (collagen–PEG, gelatin–PCL)	Blending or grafting, combining natural biocompatibility with synthetic strength	Dual biological–mechanical functionality with uniform AgNP anchoring	Enhances antibacterial and osteogenic response	Complex fabrication and interfacial instability	Collagen–PEG–Ag and gelatin–PCL–Ag scaffolds for bone regeneration	[466,467]
**Carbon-based framework** (CNTs, graphene oxide, carbon dots)	Surface functional groups nucleating or adsorbing AgNPs to form conductive hybrids	π-conjugated surfaces enabling uniform dispersion and electrical conductivity	Exhibits antibacterial, antioxidant, and sensing capabilities	Aggregation and oxidative instability under physiological conditions	CNT–Ag and GO–Ag composites for electroactive dressings and biosensors	[468,469]
**Ceramic composite** (CaP, TiO_2_, SiO_2_)	Sol–gel or co-precipitation incorporating AgNPs within inorganic lattices	Stable crystalline anchoring and slow Ag^+^ diffusion for long-term activity	Provides antimicrobial efficacy and osteoconductivity	Brittleness and porosity-dependent ion release	TiO_2_–Ag and CaP–Ag coatings for orthopedic and dental implants	[470,471]
**Metallic substrate** (Ti, stainless steel, Mg)	Surface activation followed by reduction or electrodeposition anchoring AgNPs	Micro/nano-roughened interfaces providing strong adhesion	Offers durable antibacterial performance on implant surfaces	Risk of galvanic corrosion and altered mechanical integrity	Ti–Ag and Mg–Ag coatings for orthopedic and dental applications	[472,473]
**Porous and hybrid framework** (MOFs, zeolites, silica aerogels)	Encapsulation or ion exchange retaining AgNPs within hierarchical pores	High surface area allowing sustained Ag^+^ release and adsorption capacity	Provides long-term antimicrobial and catalytic function	Complex synthesis and Ag^+^ leakage in ionic environments	MOF–Ag and zeolite–Ag composites for controlled-release coatings	[474,475]

## 4 Matrix-driven stimuli-responsive and smart silver nanocomposites

Silver nanocomposites sense local stimuli and control silver activity with spatial and temporal precision. Stimuli, endogenous (pH, redox, enzymatic activity) and exogenous (light, magnetic field, ultrasound), are implemented via matrix chemistry and nanoarchitecture to regulate Ag^+^ flux, activate photocatalysis or magnetothermal heating, and support reuse with low leaching. The unifying design principle is regulated Ag^+^ transport coupled to stimulus-activated interfacial reactivity, enabling site-specific, time-controlled activity with minimal off-target release. Representative platforms for each trigger, the underlying mechanism, and target indications are summarized in Table 2.

**Table 2:** Stimuli-responsive silver nanocomposites: Triggers, mechanisms, and targeted applications. Organized by the environmental cues that control release (pH, redox, enzymatic, photonic, magnetic, and mechanical), this table outlines the underlying mechanisms, representative materials and therapeutic applications of responsive silver systems. It highlights the versatility of trigger-controlled Ag^+^ and co-payload release strategies in precision medicine.

Stimulus type	Activation mechanism	Representative system	Biomedical function	Challenge or limitation	Representative application	Reference
**pH-responsive**	Protonation or deprotonation of functional groups modulating Ag^+^ solubility	Chitosan–Ag hydrogels and polyaniline–Ag films	Enables pH-triggered Ag^+^ release in acidic microenvironments	Narrow pH range and limited response in deep tissue	pH-responsive antibacterial dressings and coatings	[244]
**Enzyme-responsive**	Enzymatic cleavage of peptide or polysaccharide linkers releasing AgNPs	Gelatin–Ag nanocomposites with collagenase-sensitive linkers	Provides targeted antimicrobial action at infection sites	Variable enzyme expression across tissues	Protease-degradable Ag hydrogels for chronic wounds	[476,477]
**Thermal-responsive**	Polymer phase transition altering AgNP diffusion	PNIPAM–Ag nanogels	Enables temperature-controlled antimicrobial and drug release	Limited stability under physiological fluctuations	Thermo-responsive wound-healing films	[478,479]
**Photo-responsive**	Light exposure inducing plasmonic heating or photocatalytic Ag release	TiO_2_–Ag and GO–Ag nanohybrids	Provides on-demand antimicrobial activation with minimal systemic exposure	Shallow light penetration in tissue	UV/visible-triggered antibacterial coatings and photothermal therapies	[475,480]
**Magnetic or electric stimuli**	Field exposure driving particle motion or electro-redox cycling of Ag^+^	Fe_3_O_4_–Ag and PCL–Ag–graphene films	Enables remote-controlled antibacterial activity and biofilm disruption	Equipment dependence and local heating	Magneto-responsive antibacterial composites and electrodes	[481,482]
**Ultrasound-responsive**	Acoustic cavitation enhancing AgNP dispersion and ion release	PLGA–Ag microbubbles and collagen–Ag sonogels	Provides synergistic antibacterial effect with mechanical microstreaming	Limited targeting and possible tissue heating ROS and Ag^+^ action	Ultrasound-activated antibacterial scaffolds	[483]

### 4.1 pH-responsive release

Chronic wounds are mildly alkaline (∼pH 7–9), whereas intact skin is mildly acidic (∼pH 4.5–6); effective pH-responsive designs therefore remain quiescent on peri-wound skin and increase silver activity in the wound bed [[235], [236], [237]]. Beyond carboxylated gels, halloysite-supported silver in chitosan sponges provides mesoporous reservoirs that blunt burst release and shift output upward as pH rises, while maintaining fibroblast compatibility [238]. Ion-exchange carriers such as Ag-exchanged clinoptilolite use the exact gating mechanism for Ag^+^ release through counter-ion exchange that accelerates under neutral–alkaline, chloride-rich exudate, sustaining antibiofilm action with low leaching [[239], [240], [241]]. pH-switchable polymer brushes, such as surface-grafted poly(2-vinylpyridine) (P2VP), collapse when protonated and open as pH increases, reversibly exposing immobilized Ag for on–off dosing without particle loss. Because chloride rapidly converts surface Ag^0^ to AgCl, and proteins can either passivate or promote dissolution depending on affinity, benchmarking in wound-mimicking media (chloride, bicarbonate, albumin) is recommended [242,243]. For example, P2VP–Ag systems achieved greater than 95 % bacterial reduction at pH 9, while maintaining greater than 90 % fibroblast viability. In *ex vivo* porcine skin models, pH-triggered AgNP hydrogels reduced *S. aureus* loads by 3 log units within 48 h [244,245].

pH-responsive designs could also be leveraged for Ag-based therapy in certain pathological conditions that feature acidic microenvironments. For example, many solid tumors exhibit an extracellular pH between 6.2 and 6.9 due to anaerobic metabolism and lactic acid accumulation, while infection sites such as dental caries and gastric ulcers can also present low pH values [246]. Acid-responsive nanostructures, such as AgNPs encapsulated in poly(L-histidine)-coated liposomes, pH-labile hydrazone-linked polymer micelles, or mesoporous silica nanoparticles with acid-cleavable gates, have been reported to achieve selective Ag^+^ release in acidic environments, thereby enhancing therapeutic targeting while reducing off-target effects.

### 4.2 Redox-responsive systems

Reductive and oxidative gradients in diseased tissue, typically in the range of 1–10 mM intracellular glutathione (GSH), 1–50 μM extracellular thiols, and elevated H_2_O_2_ in inflamed loci, can be translated into transport and catalytic control of silver. In tumor microenvironments, where GSH concentrations can be several times higher than in normal tissue, redox-responsive Ag-based systems offer targeted activation potential [[247], [248], [249]]. Diselenide-bridged mesoporous organosilicas cleave at lower redox potentials than disulfides and, when loaded with Ag and a co-antimicrobial such as tobramycin, open their pore network under GSH/ROS conditions to accelerate release at biofilm or tumor interfaces while maintaining basal output at a low level [250,251]. Prussian blue–silver (PB–Ag) hybrids couple the reversible Prussian Blue (PB)/Prussian White (PW) redox couple with Ag to shuttle electrons and sustain ROS-mediated killing under low-intensity visible/NIR light, reducing the Ag loading required for potency [252,253]. Nanozyme-assisted constructs, such as Fe_3_O_4_@MoS_2_–Ag, impart peroxidase-like activity to convert wound-relevant H_2_O_2_ into •OH and pair it with Ag^+^/photothermal stress. The magnetic core enables recovery and reuse, thereby lowering cumulative Ag emission. In murine tumor models, redox-sensitive AgNPs coated with GSH-cleavable PEG have demonstrated selective accumulation and a greater than 70 % reduction in tumor volume within 14 days, while sparing healthy tissue. Diselenide linkers exhibit faster cleavage at −0.4 V compared to disulfide at −0.2 V, and PB–Ag hydrogels have demonstrated >80 % biofilm reduction in *P. aeruginosa* within 24 h, while Fe_3_O_4_@MoS_2_–Ag nanohybrids achieved complete MRSA biofilm clearance in murine wound models within 5 days [[254], [255], [256]].

### 4.3 Enzyme-responsive matrices

Protease-rich and hyaluronidase-rich microenvironments offer biochemical addressability for spatially restricted Ag delivery. Matrix metalloproteinase (MMP-2/9)-cleavable peptide shells, such as PVGLIG motifs, can encapsulate AgNPs during delivery and expose them only where the protease burden is high, increasing local adhesion and Ag^+^ flux at chronic-wound edges while reducing off-target hemolysis. Hyaluronidase-degradable hyaluronic acid carriers convert from diffusion barriers to permeable scaffolds in infected loci, switching Ag delivery on without altering bulk loading [[257], [258], [259], [260], [261]]. Tuning peptide sequence, grafting density, and crosslinking sets the cleavage threshold and release rate; selecting orthogonal triggers, such as MMP for ulcers *vs.* HAase for dental niches, aligns chemistry with pathophysiology. In dental models, MMP-cleavable AgNP hydrogels reduced periodontal pathogen counts by 90 % in 7 days. In GI infection models, hyaluronidase-cleavable AgNP capsules achieved a 2.5 log reduction in *Clostridioides difficile* populations. In murine full-thickness wound models, MMP-responsive AgNP dressings have been shown to accelerate re-epithelialization by 40 % and reduce inflammatory cytokines, such as TNF-α and IL-6, by over 50 %, further demonstrating their therapeutic potential [[262], [263], [264], [265], [266], [267]].

### 4.4 Light and photothermal control

Visible-light heterojunctions and plasmonic Ag enable activation under indoor or point-of-care illumination, whereas NIR absorbers contribute controlled photothermal gains that enhance Ag^+^ diffusion and membrane susceptibility [268]. Silver-modified bismuth oxides and halides exemplify efficient, UV-free platforms like Ag/BiVO_4_, improve charge separation, and rapidly inactivate *E. coli* under λ > 420 nm; ternary AgI/AgBr/BiOBr Z-schemes [269] harness Ag-halide plasmons for complete eradication at modest irradiance; and Ag/Bi_2_O_2_CO_3_ or Ag/AgBr/WO_3_·H_2_O extend activity to resistant strains while degrading antibiotic residues. Under 500 lx indoor light, BiVO_4_–Ag coatings reduced *E. coli* by >99 % in 1 h, while Bi_2_WO_6_–Ag films achieved 92 % MRSA eradication within 2 h in hospital-simulated lighting and degraded >85 % tetracycline in water. Comparative analyses have shown that Ag/BiVO_4_ systems offer broader visible-light absorption (up to ∼520 nm) compared to Bi_2_WO_6_–Ag (∼450 nm). Z-scheme configurations can also improve quantum efficiency and ROS yield by up to 30 % over single-component catalysts [270,271]. NIR-responsive fillers such as PDA or carbon can be added when thermal assistance is clinically acceptable. Beyond conventional visible/NIR photocatalysis, additional mechanisms relevant to nanomaterials include upconversion nanoparticles that convert NIR to shorter wavelengths for deeper tissue activation, and PDT approaches where AgNPs are conjugated with photosensitizers to enhance ROS generation upon light exposure [272,273]. *In vivo* murine wound infection studies using visible-light-activated Ag-based photocatalysts have reported >90 % reduction in bacterial load within 24 h and significant improvements in granulation tissue formation compared to untreated controls. While promising, clinical translation should account for potential phototoxicity at higher light intensities and limited tissue penetration depth for certain wavelengths, particularly in deeper infections [274,275].

### 4.5 Magnetic and ultrasound triggers

Magneto-plasmonic and piezocatalytic hybrids provide remote positioning, on-demand heating, and delivery enhancement with recovery/reuse pathways [276]. CuFe_2_O_4_@Ag [277] structures, including magnetic hollow fibers and CuFe_2_O_4_@Ag core–shells, integrate magnetic field responsiveness with silver-mediated antibacterial effects and demonstrate field-enhanced antibiofilm activity at AMF settings [278]. AMF-responsive Fe_3_O_4_@Ag [279] nanohybrids have eradicated *P. aeruginosa* biofilms by 98 % after 30 min of AMF exposure and, *in vivo* wound infection models have achieved a >90 % reduction in bacterial load with no observable tissue necrosis or systemic toxicity [[280], [281], [282]]. Under ultrasound, Ag-decorated BaTiO_3_ leverages piezocatalysis and plasmonic hot-electron injection to generate ROS and transiently open hydrogel meshes; AgNP hydrogels operating enhanced Ag^+^ release threefold, achieving complete *S. aureus* eradication in 24 h [283,284].

### 4.6 Outlook

Defining robust, quantifiable performance metrics will accelerate the clinical adoption of these targeted, on-demand Ag platforms.

Integrating these responsive designs with multifunctional platforms, as discussed in the following sections, such as those combining antimicrobial, regenerative, and sensing capabilities, could enable a seamless transition from targeted treatment to continuous monitoring and adaptive therapy, thereby bridging mechanistic control with clinical feedback systems for optimized patient outcomes.

**Fig. 6:**
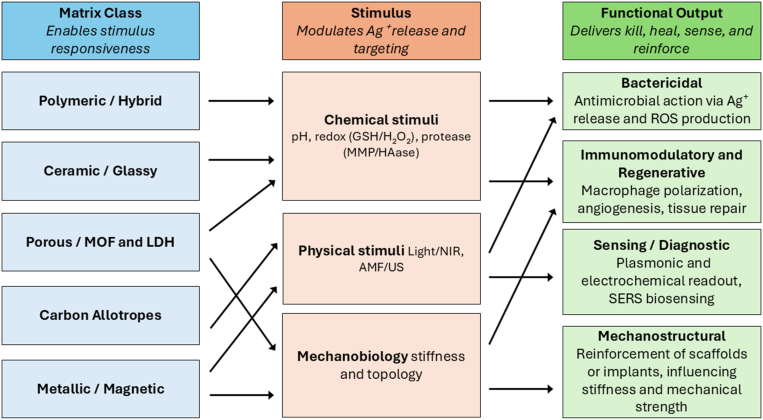
Matrix–stimulus–function design atlas for silver nanocomposites. This figure maps five distinct matrix families (i.e., polymeric/hybrid, carbon-based, ceramic/glassy, metallic/magnetic, and porous frameworks), to the typical stimuli they respond to and the functional outputs they enable. Drawing from Sections 3, 4, the matrix types are arranged to reflect how material architecture governs compatibility with environmental triggers (e.g., pH, redox, light, alternating magnetic field/AMF, ultrasound, or mechanical force) and contributes to downstream functions such as antimicrobial action, mechanical reinforcement, diagnostic sensing, and immunomodulation. This schematic diagram serves as a design-level map, highlighting how structure–function logic can be encoded through matrix selection and stimulus responsiveness. The framework supports a modular strategy for building multifunctional platforms with precise control over site-specific and application-specific outcomes.

To provide a system-level overview of how matrix design dictates silver responsiveness and functionality, Fig. 6 integrates key concepts from Sections 3, 4. This matrix–trigger–function atlas maps five primary matrix classes (i.e., polymeric, carbon-based, ceramic, metallic, and porous), to typical stimuli (e.g., pH, redox, light, magnetic fields) and downstream functional outputs (e.g., antimicrobial action, regenerative support, sensing, and mechanical reinforcement). This figure serves as a conceptual bridge between structural design and responsive behavior of silver nanocomposites.

## 5 Biointeractive silver nanocomposites

The design of biointeractive silver nanocomposites aims to address complex regenerative needs by precisely regulating Ag^+^ release to direct specific cellular responses. Hydrogels are a predominant choice because they support cell adhesion, present proteins and growth factors, retain moisture and oxygen, and enable *in situ* gelation or printing under mild conditions that preserve bioactivity. Within such matrices, adhesion motifs, cytokine-binding domains, and EVs can be incorporated to position silver within a supportive microenvironment rather than leaving it as a freely diffusing agent. Novel strategies include AgNP-loaded peptide amphiphile nanofibers that self-assemble into bioactive gels; silver–silk fibroin aerogels [285] with ultralight, high-porosity networks for advanced wound dressings; AgNPs anchored on decellularized plant vascular scaffolds offering unique anisotropic architectures; silver–bacterial nanocellulose composites doped with bioactive glass for dual regeneration and antimicrobial action; injectable AgNP-integrated cryogels with shape-memory properties for minimally invasive delivery; silver–elastin–collagen hybrids for vascular grafts; and AgNPs coupled with synthetic glycosaminoglycan mimics for targeted growth factor binding. Key parameters, dose, oxidation state, and release rate determine the balance between bactericidal efficacy and cytocompatibility. At the same time, these unique scaffold types offer distinctive structural and biological integration strategies that surpass those found in other designs [[286], [287], [288]].

### 5.1 Embedding AgNPs in extracellular matrix analogues

Embedding AgNPs into extracellular matrix (ECM) analogues enables the creation of bioactive microenvironments that closely mimic native tissue structures. Low doses of AgNPs can stimulate endothelial cell migration and vascular network formation, whereas higher concentrations may suppress angiogenesis [289]. Precise tuning of AgNP loading within ECM scaffolds, such as collagen, fibrin, or basement-membrane mimics, can therefore deliver antimicrobial protection while supporting regeneration. Unique alternatives include AgNP-integrated decellularized plant-derived vascular scaffolds for anisotropic nutrient transport, peptide-based self-assembling ECM mimics functionalized with silver for controlled angiogenesis, and ECM hydrogels crosslinked with bioactive glass particles to synergize antimicrobial and osteoconductive functions. Proteomic analyses further reveal that silver integration can modulate both pro- and anti-angiogenic signaling pathways, providing a strategic means to fine-tune vascularization dynamics precisely [290,291].

### 5.2 Hydrogels carrying EVs and AgNPs

**Fig. 7:**
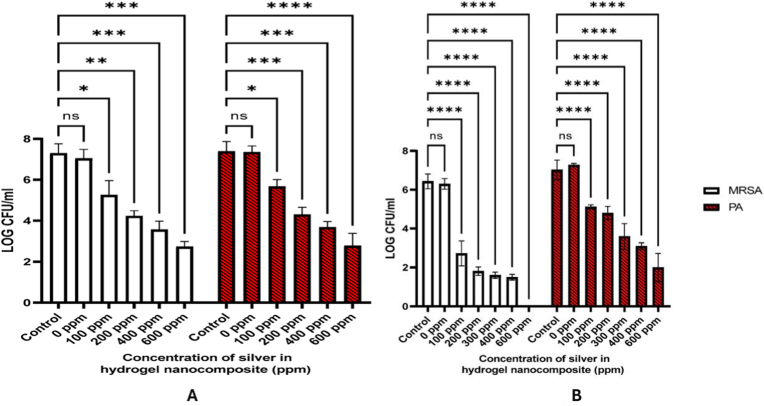

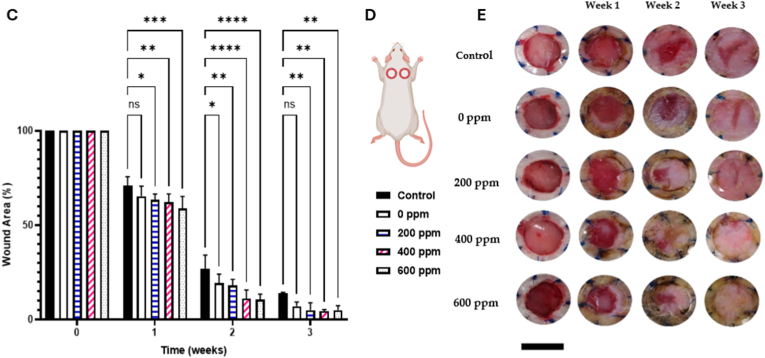

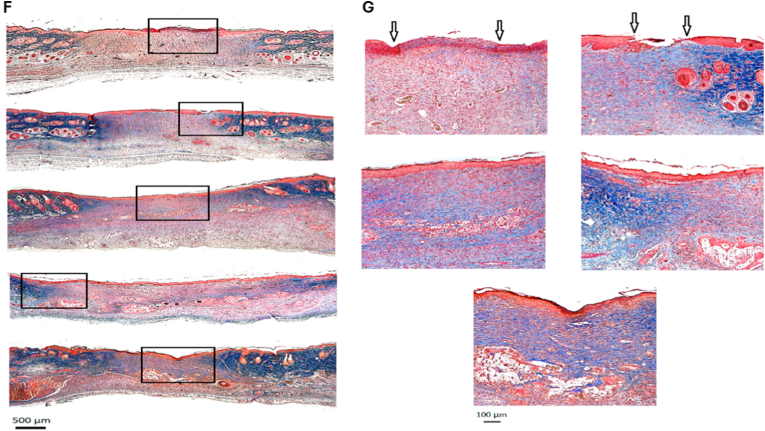
(A–B) Antibacterial activity: (A) Ag–hydrogel nanocomposite with different concentrations of AgNPs (0, 100, 200, 400, and 600 ppm) was placed in bacterial solution to be in direct contact with two common wound-infecting bacteria, MRSA and *P. aeruginosa* (PA). After 24 h, small aliquots of the bacterial solution were removed, and microorganisms were enumerated. Bacterial solution alone was considered as control. In control and hydrogel-alone (0 ppm) groups, a significant increase in bacterial number was observed after 24 h, while AgNPs significantly inhibited bacterial growth in the Ag–hydrogel nanocomposite groups. (B) The effect of Ag–hydrogel nanocomposites in preventing bacterial penetration into the hydrogel. A sterile titanium (Ti) wire disc was cast inside the hydrogels. The samples were placed in bacterial solution containing MRSA or PA. After 24 h, Ti discs were harvested, and microorganisms on their surface were enumerated. Ti disc alone in bacterial solution served as control. All Ag–hydrogel nanocomposite groups significantly inhibited bacterial penetration and reduced bacterial growth on the Ti surface. (C–E) Wound-healing study: (C) Average wound area measured over weeks 0–3. (D) Schematic image showing the location of splinted full-thickness wounds on the dorsal region of the rat. (E) Representative serial digital images of full-thickness splinted wounds in rats left untreated (control) or treated with silver–hydrogel nanocomposites containing different concentrations of silver, imaged at weeks 1, 2, and 3. (F, G) Histological analysis: (F) Histological images showing the whole wound at 2 × magnification for each treatment group. (G) Higher-magnification (10 × ) images of wound sections. Black arrows indicate areas of incomplete epithelialization. Adapted from Amiri et al., 2020, Advanced Healthcare Materials [315], under CC BY 4.0, https://creativecommons.org/licenses/by/4.0; labels were edited.

Hydrogels can be engineered to co-deliver extracellular vesicles (EVs) along with AgNPs, combining the paracrine signaling functions of EVs with silver's antimicrobial action. These hybrid systems have demonstrated enhanced angiogenesis, reduced inflammation, and faster wound closure compared with either component alone [292]. The hydrogel network protects EV cargo from degradation, enables sustained release, and supports localized delivery to target tissues. Less common innovations in this domain include thermoresponsive injectable hydrogels that release EVs and AgNPs in response to local temperature changes, click-crosslinked hydrogel systems allowing *in situ* assembly under mild conditions, and the bio-orthogonal conjugation of EVs to AgNP carriers for prolonged retention at the wound site [293,294]. An illustrative case is shown in Fig. 7, where a silver-hydrogel nanocomposite, closely aligned with emerging EV–AgNP hybrid strategies, demonstrates broad-spectrum antibacterial efficacy against MRSA and *P. aeruginosa*, prevents bacterial infiltration into tissue-mimicking substrates, and significantly accelerates wound healing *in vivo*. Complementary histological analysis confirms enhanced re-epithelialization and reduced inflammation, highlighting its potential as a multifunctional platform for translational wound care. EV–AgNP hydrogels may also increase wound closure rates in diabetic ulcer models, enhance angiogenesis, and significantly reduce inflammatory cytokines, such as TNF-α and IL-6. Incorporating microfluidic channels within the hydrogel for controlled spatial delivery of EVs and AgNPs is another emerging approach that could improve therapeutic precision [[295], [296], [297], [298]].

### 5.3 Biohybrid scaffolds and polydopamine–silver nanocomposites

Biohybrid scaffolds incorporating bioactive coatings such as polydopamine with AgNPs create multifunctional platforms for antimicrobial defense and targeted cell interaction. The polydopamine layer can reduce Ag^+^ *in situ*, conjugate bioactive ligands, and promote cell adhesion, while the silver provides antimicrobial and anticancer properties [299]. Incorporating magnetic cores enables guided positioning or retrieval under an external magnetic field. Alternative approaches could include zwitterionic [300] polymer coatings with silver for reduced protein fouling and prolonged antibacterial effects, as well as silver-functionalized conductive polymer scaffolds such as polypyrrole or PEDOT [315] for electroactive tissue interfaces. Additionally, mussel-inspired catecholamine polymers [316] with tailored degradation rates could be utilized for timed Ag^+^ release. Additional unique strategies include AgNP-loaded shape-memory [301] polymer composites capable of dynamic implant adaptation, and electrospun nanofibers of recombinant silk integrated with silver, which enhances both mechanical strength and antimicrobial performance [302].

### 5.4 Immunomodulation, angiogenesis, and tissue remodeling

Expanding the scope past antibacterial activity, biointeractive silver nanocomposites can be engineered to actively shape immune responses, promote pro-healing macrophage polarization, and regulate angiogenesis. Matrix composition, nanoparticle dosage, and release kinetics collectively influence these biological effects. Hydrogels and scaffolds incorporating specific carbohydrate motifs, bioactive peptides, or growth factor binding domains can further enhance regenerative outcomes while mitigating inflammation [303]. Unique strategies here include AgNPs combined with toll-like receptor agonists to direct innate immune pathways, silver paired with anti-inflammatory nanoparticles such as cerium oxide [304] or manganese dioxide [305] for oxidative stress mitigation, and staged-release systems that first deliver immunomodulatory or pro-angiogenic signals such as vascular endothelial growth factor (VEGF) or stromal cell-derived factor-1, before initiating antimicrobial Ag^+^ release. Additional innovative designs involve microfluidic-patterned scaffolds that enable the spatially controlled release of Ag^+^ and immunomodulators, as well as magnetically responsive composites for on-demand immune modulation. AgNP–cerium oxide composites have been shown to reduce ROS levels, increase the rate of M2 macrophage polarization, and accelerate collagen deposition. AgNP–VEGF systems also have demonstrated an increase in capillary density, underscoring their potential to coordinate inflammatory resolution, angiogenesis, and tissue remodeling [306].

## 6 Mechanobiology and functional interfaces

**Fig. 8:**
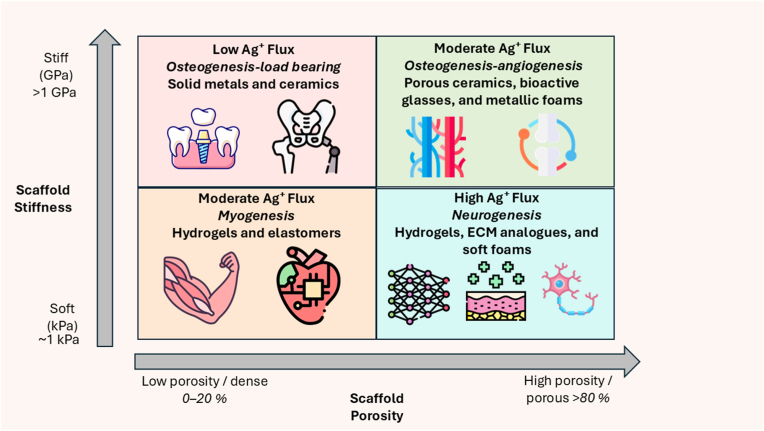
Mechanobiology-informed silver scaffold design: linking matrix cues to cellular response and therapeutic outcomes. This schematic diagram illustrates how mechanical, structural, and interfacial cues in silver nanocomposite scaffolds influence cell fate, immune response, and Ag^+^ transport, thereby coordinating antimicrobial and regenerative outcomes. Matrix stiffness and elasticity guide lineage commitment of stem cells, with soft matrices promoting neurogenesis, intermediate stiffness supporting myogenesis, and stiff matrices favoring osteogenesis. Surface topography (e.g., micro- or nano-patterning) and porosity influence cell adhesion, infiltration, and immune cell polarization. For example, rough or aligned surfaces modulate focal adhesion formation, while porous architectures enhance immune modulation and vascular integration. Stiffness and crosslinking density also modulate Ag^+^ release kinetics: soft or loosely crosslinked scaffolds allow burst release, while stiff scaffolds ensure sustained ion delivery. These physical cues integrate into multifunctional scaffold platforms that simultaneously support tissue regeneration, exert antimicrobial effects via controlled silver release, and modulate inflammation through mechanotransduction-regulated immune pathways (e.g., M1/M2 macrophage polarization).

Cells sense stiffness, elasticity, topography, and porosity through mechanotransduction, making it essential for scaffold mechanical microenvironments to guide migration, infiltration, and lineage commitment while coordinating Ag^+^ delivery. Classic stiffness benchmarks from mesenchymal stem cell studies indicate that soft, brain-like matrices promote neural differentiation, intermediate stiffness like muscle supports myogenesis, and stiff substrates encourage osteogenesis [307]. These target ranges guide material selection, crosslinking density, and scaffold architecture, while antimicrobial activity depends on the controlled release of Ag^+^. In silver-based systems, tuning the stiffness to match the target tissue enhances vascularization and integration, while topographical order influences focal adhesion maturation and traction forces [308]. To translate mechanobiological principles into functional design strategies, scaffold architectures must integrate stiffness, porosity, and surface topography with Ag^+^ transport mechanisms. Fig. 8 and Table 3 summarize these design relationships, highlighting how mechanical cues can be programmed to align with therapeutic goals, such as regeneration, immune modulation, and infection control. The following sections integrate these principles with ion-release control strategies.

**Table 3:** Mechanobiology-informed multifunctional silver scaffolds: Linking mechanics, integrated functions and biological outcomes. This table presents data on various scaffold platforms, from nanofibers and hydrogels to 3D-printed constructs, highlighting their mechanical properties, combined antimicrobial and regenerative functions, and mechanobiological effects on cells. It offers a roadmap for designing constructs that utilize mechanical cues to promote wound healing.

Scaffold platform	Mechanical property	Integrated function	Biological response	Limitation	Application	Reference
**Electrospun PCL–Ag nanofiber**	Tensile modulus 5–10 MPa; flexible and porous	Sustained Ag^+^ release with topographical cues	Promotes fibroblast migration and antibacterial protection	Limited load-bearing capacity	Soft-tissue regeneration and wound closure	[123,465]
**Gelatin–Ag cryogel**	High compressibility and interconnected pores	Incorporation of AgNPs within elastic 3D networks	Enhances angiogenesis and infection control	Rapid degradation and low strength	Injectable wound-healing scaffolds	[484,485]
**Silk fibroin–Ag composite**	Tunable stiffness 100 kPa–1 MPa	Embedding AgNPs in β-sheet domains	Supports osteoblast differentiation and antibacterial activity	Processing complexity and brittleness	Bone-tissue engineering scaffolds	[486,487]
**PCL–HA–Ag hybrid scaffold**	Compressive modulus >20 MPa	Combination of HA osteoconductivity and AgNP antibacteriality	Provides dual antibacterial and osteogenic function	Interfacial mismatch and uneven Ag distribution	Load-bearing bone graft substitutes	[488,489]
**PDMS–Ag flexible substrate**	Elastic modulus 1–2 MPa; stretchable	Conductive surface with embedded AgNPs	Enables antibacterial smart sensors and bioelectronic interfaces	Possible Ag^+^ leaching under cyclic strain	Wearable antibacterial electronics	[490,491]

### 6.1 Mechanical properties of chitosan–silver nanofibers

Electrospun chitosan blended with PEO or PVA forms ECM-like mats that incorporate AgNPs, exhibiting tensile strengths within the physiological range of the skin. These scaffolds exhibit potent antibacterial activity against *P. aeruginosa* and *S. aureus*, while maintaining high viability of keratinocytes and fibroblasts. Co-loading therapeutic agents such as curcumin, sulfanilamide, or chondroitin-4-sulfate introduces anti-inflammatory and pro-healing effects without compromising mechanical integrity. Their high porosity, fiber diameters, and a controlled burst and sustained Ag^+^ release profile facilitate gas exchange, early bacterial load reduction, and prolonged antimicrobial protection. Mechanistic insights indicate that surface-bound AgNPs dominate the initial release phase, while the sustained phase is governed by polymer matrix degradation and diffusion. Comparable systems include silk-fibroin/Ag nanofibers with enhanced mechanical strength and pH-responsive drug delivery, and bacterial-cellulose/Ag membranes characterized by high water content, optical transparency, and uniform AgNP dispersion, offering durable antibacterial performance while retaining structural stability.

### 6.2 Topographical and hierarchical features

Nanoscale architecture critically regulates cell adhesion and force generation with fiber alignment, diameter, and surface roughness tailored to direct cellular contact guidance and migration while enabling sustained delivery of bioactive agents [309]. Electrospun PVA mats [310] embedded with AgNPs and dendronized endolysin preserve enzymatic activity within high-surface-area fiber networks, ensuring combined bactericidal and enzymatic functions. Layer-by-layer [311] deposition and electrospraying techniques enable the creation of AgNP gradients or spatial patterns on pre-formed fibers, allowing for the localized modulation of adhesion strength and ion release. Hierarchical surface designs of particular note include AgNP-integrated graphene oxide scaffolds, which combine high surface area with electrical conductivity to promote cell signaling while delivering antibacterial activity; Ag-functionalized nanoridge patterns on polymer substrates, such as polydimethylsiloxane or polycaprolactone, which leverage engineered topography for both antimicrobial and regenerative outcomes; and honeycomb bilayer nanotube templates with uniformly distributed small-diameter AgNPs that provide sustained bacteriostasis and promote osseointegration [312].

### 6.3 Coupling mechanics with Ag^+^ delivery

In AgNP-loaded fibers and hydrogels, initial burst release is primarily dictated by surface-exposed particles, while polymer network diffusion, swelling dynamics, and degradation rates control sustained ion delivery. Crosslinking density thus serves as a dual regulator of mechanical stiffness and Ag^+^ release kinetics [313]. Polyacrylamide/chitosan hydrogels are synthesized via γ-irradiation to integrate robust network reinforcement with *in situ* Ag^+^ reduction to metallic silver, while polysaccharide-reduced hydrogels prepared through hydrothermal methods achieve uniform AgNP dispersion and tunable mechanical properties. Additional designs include mesoporous bioactive-glass/poly(L-lactic acid) (PLLA) scaffolds providing controlled release with high porosity for cellular infiltration, and bacterial-cellulose/Ag hydrogels maintaining stable surface-bound AgNPs for prolonged antibacterial activity. Sr/Ag-modified calcium-phosphate coatings exemplify a chemical tuning strategy for modulating dissolution-driven Ag^+^ release while preserving strong adhesion to metallic substrates [[314], [315], [316]].

### 6.4 Toward mechanobiology-informed implant design

The design of mechanobiology-informed implants focuses on particular nanomaterials and composite systems that turn mechanobiological principles into functional designs. Metallic-glass Ti_3_Au alloys doped with trace Ag/Cu achieve significant hardness, integrating exceptional mechanical strength with rapid antibacterial performance and cytocompatibility. As a soft, regenerative counterpart, self-healing AgNP–exosome hydrogels integrate anti-inflammatory action with pro-angiogenic support, aligning their compliance with vascular and neural repair needs. For load-bearing bone applications, alternative options to the systems mentioned earlier include Ag-doped zirconia-toughened alumina [317,318] composites designed for high strength and wear resistance in joint implants; Ag-functionalized bioresorbable magnesium alloys [319] that degrade at the site of action while releasing antibacterial ions; or nanostructured Ag-bioactive glass [320] coatings engineered for both osteoconductivity and infection prevention [321].

Ag-functionalized poly(ether ether ketone) (PEEK) [322] surfaces maintain cortical bone modulus while imparting bacteriostatic function. Together, these examples demonstrate how mechanical compatibility, topographical control, and precisely tuned Ag^+^ release can be strategically combined to meet the specific biological and functional demands of targeted tissues.

## 7 Multifunctional silver nanomaterials

Silver nanomaterials have emerged as versatile platforms that integrate structural, electronic, and immunological functionalities. Their multifunctionality enables desired interaction with both biological systems and technological interfaces, expanding silver's role across diverse scientific and engineering domains.

### 7.1 Bioelectronic and cyber-physical interfaces

**Fig. 9:**
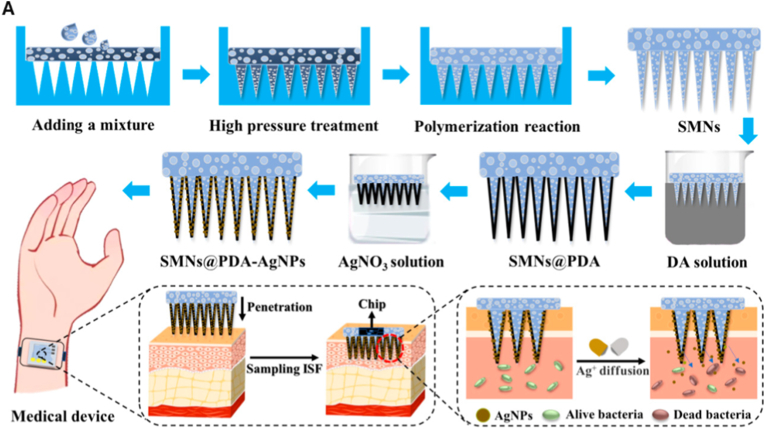

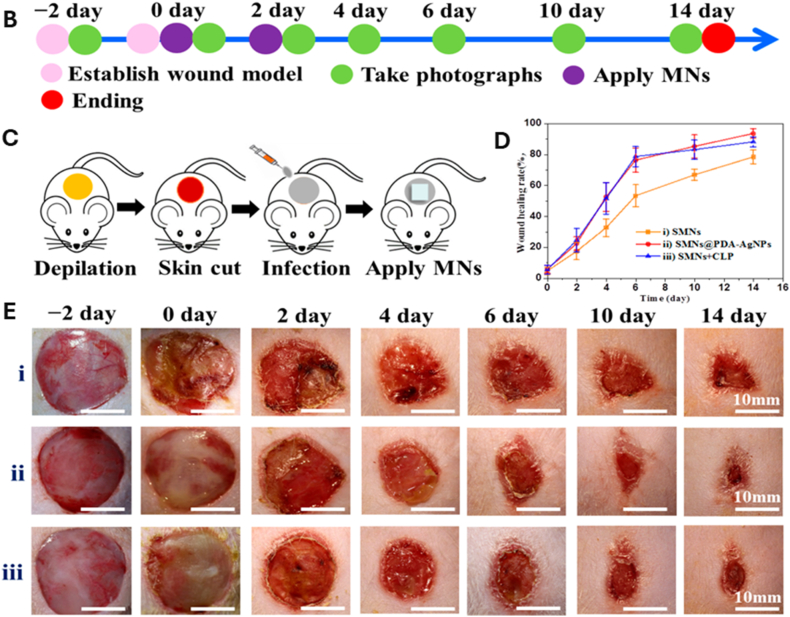

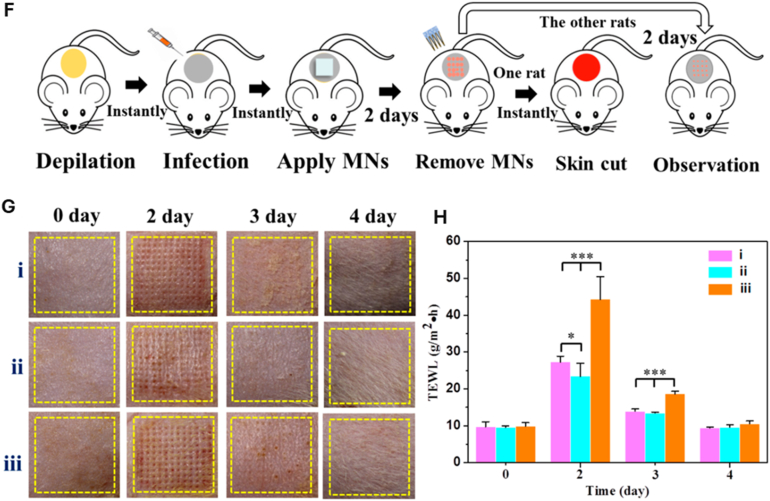

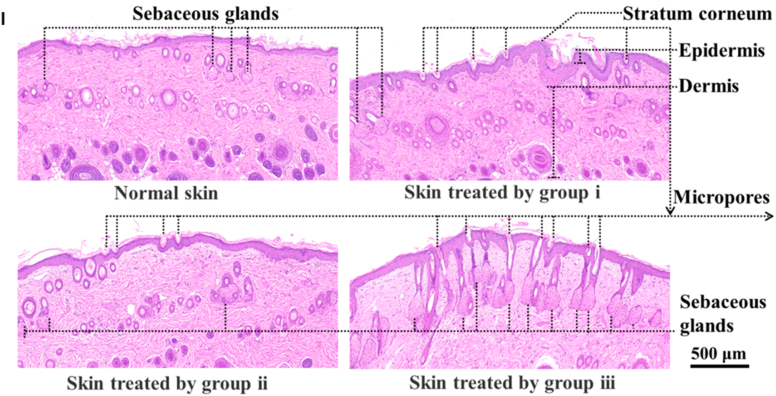
(A) Scheme of the fabrication and application of SMNs@PDA-AgNPs for medical devices. (B–E) Effects of MNs on wound healing: (B) Scheme of the experimental procedures. (C) Scheme illustrating the experimental sequence for depilation, skin cutting, infection, and MN application. (D) Wound-healing rates and (E) photographs of wound healing of rats receiving different treatments: (i) SMNs, (ii) SMNs@PDA AgNPs, and (iii) SMNs + CIP. (F–H) Antibacterial activity of MNs during application: (F) Scheme of the experimental procedures. (G) Recovery process of micropores created by MNs: (i) SMNs without bacterial infection, (ii) SMNs@PDA-AgNPs with bacterial infection, and (iii) SMNs with bacterial infection. (H) Trends in changes of TEWL during micropore healing. Abbreviations: SMNs: silk microneedles; MNs: microneedles; CIP: ciprofloxacin; TEWL: transepidermal water loss. Adapted from Zhang et al., 2023, Int J Pharm [353], under CC BY 4.0, https://creativecommons.org/licenses/by/4.0; labels were edited.

Silver's exceptional conductivity positions it as a natural mediator between antimicrobial scaffolds and electronic circuits [323]. Unlike conventional conductive polymers or carbon-based materials, silver simultaneously suppresses microbial colonization while transmitting bioelectrical signals, enabling its use in wound-care patches, neural probes, and wearable bioelectronic devices. Recent prototypes of silver nanowire mesh embedded in elastomeric hydrogels demonstrate dual capacity for continuous electrophysiological monitoring and ion release-based disinfection, opening the door to “electronic dressings” that promote healing while sensing [[324], [325], [326]]. A novel example of a multifunctional cyber-physical wound care system is shown in Fig. 9, where PDA–silver-coated sponge microneedles (SMNs@PDA–AgNPs) combine minimally invasive delivery with localized antimicrobial activity and accelerated healing outcomes. The microneedles not only inhibit MRSA infiltration and preserve tissue integrity but also enable interstitial fluid sampling—an essential step toward real-time diagnostic feedback in smart wound dressings [327].

Recent work by Jiang and colleagues exemplifies this potential: they developed a temperature-responsive hydrogel dressing composed of poly(acrylic acid)-grafted poly(N-isopropyl acrylamide), polyacrylamide, and a 3D network of silver nanowires. The Ag nanowires [328] imparted both antibacterial activity and electrical conductivity, while the hydrogel matrix ensured softness and adhesion [329]. Such designs suggest a cyber-physical future in which bioelectrical signals and pathogen-triggered Ag^+^ release are coordinated via wireless feedback loops. To realize these systems clinically, key challenges remain maintaining conductive integrity under strain and swelling, regulating Ag^+^ release within safe limits during electrical stimulation, and ensuring data security [330]. When paired with wireless modules and cloud-linked analytics, these systems could extend silver nanocomposites into the field of digital health, forming closed-loop care cycles where physiological data trigger adaptive antimicrobial or electrostimulation responses. This cyber-physical intersection establishes silver as more than a therapeutic agent; it becomes a node in patient–device–data ecosystems.

### 7.2 Immunoengineering and adjuvant design

Silver nanomaterials possess immunoactive properties that can be harnessed to modulate both innate and adaptive immune responses. At controlled doses, silver composites influence macrophage polarization toward reparative phenotypes and enhance dendritic cell antigen presentation, suggesting potential as localized vaccine adjuvants. Studies have shown that AgNPs can promote endothelial migration and monolayer formation in lower doses, whereas higher doses inhibit angiogenesis, emphasizing the importance of dose-dependent immunomodulation [[331], [332], [333]]. Embedding silver in immunoactive scaffolds may therefore provide a dual benefit: preventing peri-implant infection while simultaneously priming protective immunity against resident pathogens. In high-risk surgical or transplant contexts, such scaffolds could train the local immune environment for tolerance or pathogen-specific memory, thereby reducing reliance on systemic antibiotics or immunosuppressants, thus repositioning silver as a mediator of immune programming [334].

### 7.3 Hybrid living architectures

A frontier concept lies in the integration of silver into engineered living materials, where synthetic biology and nanotechnology converge. By embedding AgNPs into bacterial cellulose, yeast-derived matrices, or cell-laden three-dimensional (3D) bioprinted constructs, these composites can unite microbial resistance, electrical conductivity, and regenerative potential within a single dynamic architecture [[335], [336], [337]]. In such “symbiotic composites,” the inorganic silver phase provides resistance to infection and electroactive cues, while the living phase adapts, remodels, and promotes tissue integration [338]. Recent approaches have further advanced this vision by demonstrating programmable functionalities where silver not only imparts antimicrobial and conductive properties but also enables responsive behavior. Unlike biointeractive hydrogels that merely release signaling vesicles, these hybrid living systems actively evolve in response to their environment, engaging in mutual feedback between cellular and material components. The result is a biohybrid platform that not only integrates with host tissue but also senses, adapts, and regenerates over time while preserving sterility [339]. Such constructs herald a shift toward next-generation programmable implant devices that are not static but symbiotic, capable of dynamic remodeling and physiological integration, bridging the boundary between engineered systems and living tissue.

### 7.4 Structural and systemic integration

Silver's multifunctionality also extends to structural engineering. Incorporating silver directly into load-bearing polymers, fibers, or metallic backbones creates “mechanical–biological hybrids” capable of coupling structural reinforcement with biological safety (Representative platforms and their mechanics–function relationships are summarized in Table 3). Orthopedic fixation devices fabricated from silver-reinforced polymers are one example of these structures; they bear physiological loads while resisting biofilm formation, addressing two traditionally opposing design goals in a single construct [340,341]. Beyond orthopedics, integrating silver into vascular stents or sutures presents opportunities for devices that maintain mechanical integrity under hemodynamic stress while simultaneously mitigating the risk of infection [342,343]. This perspective elevates silver from a coating applied post-fabrication to a core structural constituent in biomedical device engineering.

### 7.5 Environmental and societal coupling

In wearable devices, silver composites can simultaneously inhibit skin pathogens, monitor perspiration biomarkers, and transmit data to cloud-based health systems, forming triple-function platforms where hygiene, diagnostics, and telemedicine converge. For instance, AgNP-doped hydrogels have been employed in epidermal sensors to detect sweat metabolites while actively preventing skin infections, highlighting the potential for personal health monitoring systems that integrate microbial protection with physiological readouts [344,345]. In clinical environments, silver-based wall coatings and air-filter membranes could couple antimicrobial control with real-time monitoring of airborne pathogens. Embedding Ag-decorated aerogels or Ag-oxide/silica membranes into hospital air-handling or potable water systems has demonstrated catalytic degradation of organic contaminants along with broad-spectrum antibacterial efficacy. These multifunctional systems not only disinfect but also offer a platform for detecting pathogens and pollutants, linking environmental safety to healthcare infrastructure [[346], [347], [348]]. Wastewater-embedded silver composites could further integrate disinfection with contaminant sensing, bridging environmental stewardship and public health. As telemedicine and ecological sensing technologies continue to converge, silver nanocomposites may become central components in networks that unify individual health, hygiene, and ecological oversight [[349], [350], [351]]. To support long-term deployment, sustainable design principles, including the recovery and recycling of silver at end-of-life, will be crucial in mitigating environmental accumulation and ensuring responsible use.

### 7.6 Translational benchmarks for multifunctionality

To progress from laboratory prototypes to clinical and societal deployment, silver nanocomposites must meet clear, quantitative criteria. First, antimicrobial efficacy should be maintained under mechanical deformation, electrical bias, or environmental exposure, with Ag^+^ flux reported in standardized units (e.g., μg cm^−2^ over time) and aligned with cytocompatibility thresholds. Second, secondary functions, such as electrical conduction, structural reinforcement, immune modulation, or biosensing, must be independently validated and shown to operate in parallel without functional interference. Third, durability must be demonstrated across multiple activation cycles, including stretching, photothermal heating, or magnetic actuation, with stable performance over time. Fourth, manufacturing pathways should be scalable, reproducible, and regulatory-compliant, with rigorous characterization of particle size distribution, surface chemistry, and ion-release kinetics [352,353]. Meeting these standards requires not only material innovation but also cross-disciplinary validation in clinically relevant environments. Establishing multifunctionality as a measurable and reproducible performance category will be central to advancing silver nanocomposites from experimental composites to key platforms in biomedicine, environmental sensing, and wearable technology. These translational benchmarks provide both a roadmap for researchers and a framework for regulators to assess next-generation silver-based materials.

## 8 Data-driven design and multi-omics for safe-by-design silver nanocomposites

**Fig. 10:**
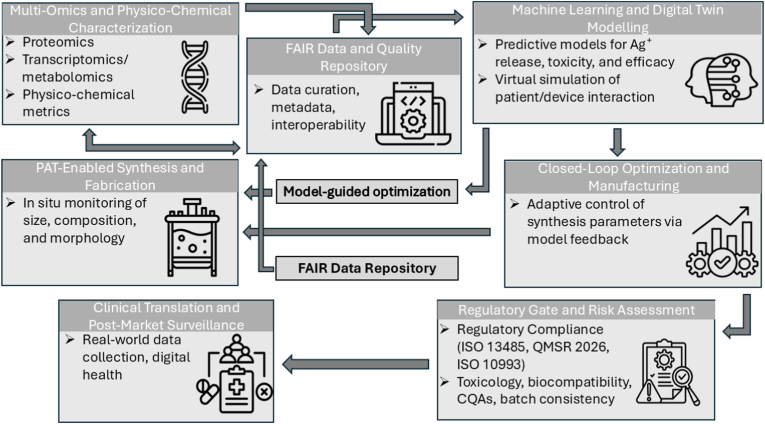
Safe-by-design (SbD), data-integrated development pipeline for silver nanocomposites. This schematic diagram outlines a closed-loop, AI-augmented framework that integrates synthesis, characterization, modeling, and regulatory alignment to accelerate the design of safe and effective silver nanocomposites. Flow synthesis platforms are coupled with process analytical technologies (PATs) for real-time control of material fabrication parameters (e.g., particle size, silver loading). Multi-omics characterization (e.g., transcriptomics, proteomics, metabolomics) links nanomaterial features to biological responses. FAIR (Findable, Accessible, Interoperable, Reusable) data infrastructures standardize experimental outputs for sharing and reanalysis. Machine learning and digital twins predict structure–function relationships and optimize synthesis pathways. Regulatory checkpoints are embedded as feedback gates, aligning iterative design with safety thresholds and quality-by-design (QbD) principles. The pipeline emphasizes continuous feedback among materials science, biological testing, and computational modeling, enabling anticipatory SbD strategies that reduce clinical translation risk while improving reproducibility and performance.

Designing clinically credible silver nanocomposites means relating synthesis parameters to structure, ion release, biological response, and performance under realistic conditions. Empirical trial and error are too slow to navigate this complex design space. To operationalize predictive SbD strategies, Fig. 10 presents a closed-loop development framework integrating flow synthesis, process monitoring, multi-omics, FAIR data, and AI-guided modeling, enabling continuous optimization and early regulatory alignment [354]. FAIR data infrastructures further accelerate this shift from reactive testing to anticipatory design [355]. Table 4 summarizes the SbD levers, regulatory checkpoints, and clinical translation pathways.

**Table 4:** Data-driven and SbD approaches: Methodologies, outputs, and implementation considerations. This table summarizes high-throughput combinatorial synthesis, machine learning models, federated and transfer learning, multi-omics integration, and digital twin technologies. It shows how advanced computational and analytical tools guide the rational design and safety assessment of silver nanocomposites. It highlights the emerging synergy between data science and materials engineering.

Approach or tool	Methodological basis	Outputs or predictions	Advantage	Limitation	Translational perspective	Reference
**Molecular-dynamics simulation**	Atomistic modeling of Ag–matrix interactions and surface reactivity	Prediction of Ag^+^ release kinetics and binding energy landscape	Provides mechanistic insight into stability and ion flux	Computationally intensive and limited timescale	Guides material selection and interface design	[492,493]
**Density-functional theory**	Quantum-mechanical modeling of Ag–ligand interactions	Evaluation of charge transfer and reactive sites on nanostructures	Enables atomic-level understanding of antibacterial mechanisms	High computational cost and simplified assumptions	Supports catalyst design and biocompatibility analysis	[494,495]
**Machine-learning model**	Regression or classification trained on experimental datasets	Prediction of antibacterial efficiency, toxicity, and Ag^+^ release	Accelerates optimization and safe-by-design screening	Dependent on the dataset quality and interpretability	Enables rapid design of low-toxicity Ag composites	[496,497]
**QSAR modeling**	Correlation of AgNP descriptors with biological activity	Identification of key physicochemical predictors of cytotoxicity	Supports regulatory risk assessment	Requires standardized descriptors and large datasets	Facilitates predictive toxicity modeling	[498,499]
**Digital-twin and multiscale modeling**	Integration of experimental, omics, and simulation data	Real-time prediction of performance and safety	Bridges bench-to-bedside translation via adaptive refinement	Complex data integration and validation needs	Forms foundation for adaptive safe-by-design frameworks	[26]

### 8.1 Artificial intelligence and machine learning for synthesis optimization and safety assessment

Machine learning is emerging as a practical engine for safe-by-design silver nanocomposites by mapping synthesis variables directly onto physicochemical attributes and biological outcomes. A landmark analysis of literature-curated AgNP datasets demonstrated that supervised models not only predict particle size and antibacterial potency, but also critical factors, such as synthesis time, reaction scale, and capping chemistry, that often elude human intuition [356]. These insights have paved the way for closed-loop platforms that combine Bayesian optimization with droplet-based microfluidics to iteratively converge on target plasmonic absorbance spectra and nanoparticle morphologies under 150 iterations [[357], [358], [359]]. Inline optical readouts guide real-time reagent modulation, while integration with digital twin simulations enables dynamic control of nucleation, coating density, and ion-release kinetics, addressing batch reproducibility [360,361].

Next-generation models harness graph neural networks that encode nanoparticle structure, protein corona composition, and dissolution kinetics to predict cellular uptake, circulation half-life, and antimicrobial efficacy with greater accuracy than single-modality learners [362,363]. Transfer learning, data augmentation, and ensemble modeling counter data scarcity and improve model robustness across diverse nanoparticle families [364,365]. Federated learning architectures [366] further enable these predictive models to be trained on distributed datasets, including GO–Ag composites, nanowire hydrogels, and mesoporous Ag@MOFs, without requiring the exchange of raw data, thereby preserving confidentiality while enhancing generalizability.

On the safety front, nanomaterial quantitative structure–activity relationship (nano-QSAR) frameworks [367,368] link Ag^+^ release rates in physiologically relevant thiol- and chloride-rich environments, zeta potential at biological pH, and corona fingerprints (e.g., apolipoproteins, complement factors) to oxidative stress, genotoxicity, and hemocompatibility endpoints. These interpretable models act as early safety screens, reducing reliance on costly *in vivo* assays and aligning the field with the Safe- and Sustainable-by-Design (SSbD) regulatory paradigm. These advances move silver nanocomposite development from empirical trial-and-error toward a reproducible, mechanistically grounded, and regulatory-ready discipline, positioning machine learning as a cornerstone in the rational design of multifunctional, clinically translatable materials [369]. These design-phase AI/ML tools set the stage for AI-enabled diagnostics and decision support that couple imaging and omics to material performance.

### 8.2 AI-enabled diagnostics, modeling, and decision support

AI is redefining how silver nanocomposites are tracked, modeled, and selected for clinical and environmental deployment by unifying imaging, omics, and materials descriptors into actionable predictions. Computer-vision pipelines quantify Ag distributions in microscopy and whole-organ images at single-cell resolution, turning qualitative measures of tissue penetration and biofilm infiltration into objective covariates for modeling and feedback [370]. SERS, enabled by silver's plasmonic properties, offers a unique diagnostic interface: Ag-based substrates paired with machine-learning classifiers can distinguish pathogenic bacteria, and even differentiate methicillin-resistant from methicillin-susceptible *S. aureus* with high accuracy, providing simultaneous detection and therapeutic delivery on the same platform [371,372].

Graph neural networks integrate heterogeneous inputs, including particle structure, corona proteomes, single-cell transcriptomes, histology tiles, and exposure dose, into predictive models of cellular uptake, clearance, and immune response. These models capture complex, nonlinear dependencies, such as thiol-driven dissolution or the stabilizing effects of apolipoprotein-rich coronas, which prolong systemic circulation. On the design front, generative models propose novel nanoparticle architectures that optimize potency, release profiles, and biocompatibility. Reinforcement learning frameworks, combined with Bayesian optimization, enable autonomous reactors to adjust reaction parameters, thereby compressing development timelines from months to days [373].

These AI-driven systems establish a fully integrated, end-to-end decision-making pipeline: imaging quantifies biodistribution, omics provides mechanistic annotations, models rank SbD-compliant candidates, and bedside theranostic readouts close the loop with real-time feedback. This ecosystem elevates silver nanocomposites from opportunistic antimicrobials to predictive, regulatory-ready platforms grounded in evidence, adaptability, and automation.

### 8.3 Multi-omics to map nano–bio interactions and de-risk design

Omics technologies now make it possible to deconstruct how silver nanocomposites engage with biological systems, converting black-box outcomes into mechanistic signatures that can inform SbD development [374]. Transcriptomic and metabolomic profiling of AgNP-exposed cells consistently implicates oxidative stress responses, disruption of purine and glycerophospholipid metabolism, and the induction of cell-cycle arrest and apoptosis. Lipidomic analyses further reveal redox imbalances and mitochondrial dysfunction tightly linked to Ag^+^ release kinetics, offering pathway-level markers that distinguish antimicrobial activity from cytotoxic overload [375,376].

Proteomics [377] has shown that AgNPs rapidly acquire a protein corona in biological fluids. This corona, comprising albumin, immunoglobulins, apolipoproteins, and complement proteins, modulates hydrodynamic size, surface charge, cellular uptake, and downstream immune signaling. In experimental models, citrate-coated AgNPs exhibited reduced cellular uptake by lung epithelial and endothelial cells upon formation of a protein corona, concomitantly attenuating cytotoxicity. Furthermore, pharmacological inhibition of the scavenger receptor class B type I (SR-BI) potentiated suppression of IL-6 expression [378,379].

Temporal multi-omics profiling captures the dynamic sequence of biological responses, revealing early stress signaling followed by repair or apoptotic programs, enabling researchers to distinguish between reversible adaptation and irreversible damage. Emerging single-cell and spatial omics methods further resolve heterogeneity across tissues, mapping AgNP accumulation to specific cell types, vascular proximity, and oxygen gradients. Quantitative anchors from ICP-MS [380] and single-cell mass spectrometry [381] help align omics signatures with absolute particle burden, thereby distinguishing between internalized and surface-bound silver [382,383].

Machine-learning [384] models trained on corona fingerprints, omics profiles, and physicochemical descriptors can predict uptake, immune activation, and toxicity potential, enabling *in silico* screening of Ag formulations before clinical testing. However, the utility of these models depends on standardized sample preparation, protein depletion protocols, data annotation practices, and interoperable platforms for multi-omics fusion. By incorporating host factors such as age, sex, and genetic background, multi-omics approaches enhance generalizability and explain variability in host responses, ultimately delivering mechanistic, reproducible, and clinically translatable predictors to de-risk the design of Ag nanocomposites [385,386].

### 8.4 Closed-loop, high-throughput pipelines for Ag composite discovery

The complexity of silver nanocomposites demands workflows that can systematically explore formulation space while enforcing reproducibility. Closed-loop, high-throughput pipelines couple combinatorial synthesis with automated measurement and real-time feedback control. Microfluidic reactors and robotic liquid handlers generate large libraries under precisely controlled conditions, while inline UV–vis and Raman spectroscopy, electrochemical probes, and label-free particle analytics capture size, morphology, plasmonic behavior, and dissolution kinetics in real time [[387], [388], [389]]. Automated microfluidic reactors coupled to Bayesian optimization algorithms enable iterative AgNP production, where real-time spectroscopic monitoring informs parameter tuning until the system converges on target nanoparticle size and controlled ion-release profiles. Digital twin [390] simulations of these reactors predict nucleation dynamics and growth outcomes based on feed composition, pH, and ligand concentration, enabling informed decisions before any physical experiment is conducted, thereby minimizing waste and maximizing efficiency [391].

High-content cellular assays quantify antimicrobial activity and cytotoxicity across multiple cell types, while pooled *in vivo* screening using DNA/RNA barcoding enables simultaneous tracking of various formulations, reducing animal use while generating rich organ-level biodistribution and immune response data. This closed-loop activity mapping supports iterative design cycles: virtual screening, physical libraries, *in vivo* barcoded readouts [392], and machine learning feedback, similar to a directed evolution framework for nanomaterials [[393], [394], [395]].

Representative library formats demonstrate the breadth of multifunctional opportunities, including Ag@MOFs [396] with tunable pore-mediated ion flux, graphene–Ag hybrids [397] that balance conductivity and antimicrobial action, degradable Ag nanocages for responsive release, and Ag–cellulose or Ag–phosphate glass composites [398] for localized, sustained delivery. Importantly, such pipelines enable the deconvolution of structure-function relationships where matrix composition, corona formation, and release dynamics intersect [399]. When coupled with FAIR-compliant, machine-readable metadata, standardized proteomics workflows for corona profiling, and federated learning architectures [400] across institutions, these platforms transform high-throughput experimentation into a reproducible and regulatory-aligned engine for SbD silver nanocomposites [401,402].

### 8.5 Data standards, ethics, and translation readiness

Impactful machine learning and predictive modeling depend on curated, interoperable, and auditable data. Adhering to FAIR principles, ensuring that datasets are findable, accessible, interoperable, and reusable, is foundational for reproducibility, transparency, and regulatory acceptance. Implementing controlled vocabularies and ontologies for materials, biological media, exposure metrics, and assay endpoints, as well as assigning persistent material identifiers [e.g., European Registry of Materials identifiers (ERM IDs)], makes silver nanocomposite datasets machine-readable and cross-comparable across labs and platforms [403,404].

Harmonized reporting standards and minimum-information checklists for synthesis, characterization, and bioassays, particularly for Ag^+^ release kinetics, particle size distributions, corona proteomics, and single-cell analyses, should accompany all publications and databases. These checklists convert supplementary data into structured assets that power robust modeling and simplify regulatory review [405].

To overcome data scarcity, site bias, and fragmented repositories, collaborative data curation and repository standardization are essential. Transfer learning, ensemble methods, and federated learning enhance generalizability while protecting privacy, enabling models to be trained on clinical and industrial datasets without exchanging raw data. Explainable AI adds a critical layer of transparency, offering feature-level rationales that regulators and clinicians can interpret [[406], [407], [408]].

As silver composites move toward patient-facing applications, traceability and adaptability become key. Techniques such as molecular barcoding, isotopic tagging, and optical encoding allow *in vivo* pooling while maintaining formulation-specific resolution. Graphics processing unit (GPU)-accelerated analytics shorten the time between image or omics data acquisition and model output, enabling real-time, adaptive dosing and formulation adjustment based on interim data. Finally, ethical and sustainability considerations must be embedded into the entire development loop. SbD constraints must be enforced not only for efficacy and toxicity but also for environmental persistence and material equity [409].

## 9 Biomedical and clinical applications of silver nanocomposites

Silver nanocomposites have advanced from experimental stages to clinical applications. In various fields, both randomized trials and marketed devices now demonstrate that silver-based systems can reduce infection rates, accelerate healing, and promote tissue integration when properly designed [410].

### 9.1 Wound management

Wound care is the most developed area for silver use. Randomized and prospective studies consistently report reductions in infection and faster healing when silver dressings are applied to high-risk wounds. In a multicenter gastrointestinal surgery trial, an ionic-silver hydrofiber dressing (AQUACEL® Ag) reduced superficial surgical-site infections and improved patient comfort and healing times. In partial-thickness burns, AQUACEL® Ag outperformed silver sulfadiazine cream, lowering pain scores and reducing overall treatment costs [411,412].

Commercial dressings reflect the clinical design principles. Mepilex® Ag [413], a soft polyurethane foam with Safetac® [414] adhesive, seals wound edges to reduce maceration and allows for painless removal while initiating antimicrobial activity. Biatain® [415] Ag contains a patented silver complex in a 3D polyurethane foam, conforms to wound beds, and continuously releases Ag^+^ for up to a week; clinical series supported by the manufacturer report faster healing of infected wounds compared to other treatments. PolyMem Silver® [416] enhances this method by integrating cleansing, exudate control, inflammation reduction, and pathogen elimination into one multifunctional matrix [[417], [418], [419]].

Emerging designs are evolving from static release systems toward stimuli-responsive dressings. For example, near infrared photothermal hydrogels release Ag^+^ and generate ROS selectively at infected sites, which reduces baseline dosing and minimizes cytotoxicity. To enable meaningful comparison across different platforms, future trials should report Ag^+^ flux in chloride and thiol-rich wound fluid, hydrodynamic size, and ζ-potential in protein-rich media, as well as patient-centered outcomes such as healing rate, pain relief, and long-term recurrence of infection [[420], [421], [422]].

### 9.2 Critical-care airways: silver-coated endotracheal tubes

Ventilator-associated pneumonia (VAP) is a frequent complication in intensive care patients, and silver-coated endotracheal tubes (ETTs) have been introduced to suppress early biofilm formation. A large multicenter trial demonstrated that silver-coated ETTs halved early VAP incidence and delayed onset relative to standard tubes. Meta-analyses confirm these benefits, although effects on length of stay and mortality are modest [423,424]. Mechanistic studies show that chitosan–AgNP coatings on polyvinyl chloride decrease protein adsorption and reduce mucus buildup after 48–72 h of ventilation. Effective airway coatings must therefore combine stable silver anchoring with resistance to fouling, while maintaining controlled release under humid, protein-rich conditions. Design strategies increasingly couple ultrathin silver films with anti-fouling polymers or microstructured topographies to sustain low-level antimicrobial action. Standardized reporting of ion release in airway-simulating fluids, along with ventilation parameters and dwell times, is crucial for regulatory comparison and device benchmarking [423,425,426].

### 9.3 Orthopedic infection mitigation

Deep infection following joint replacement or oncologic limb salvage surgery remains one of the most significant challenges in orthopedics. Silver coatings on megaprostheses and trauma hardware provide prophylaxis in these high-risk contexts. Registry analyses indicate a modest absolute reduction in the risk of revision surgeries required [427]. In a pilot series of salvage arthroplasties, reinfection occurred in 40 % of silver-coated versus 57 % of uncoated implants. In European registries, MUTARS® silver-coated tumor megaprostheses [428] reduced infection rates without evidence of systemic toxicity.

Commercial technologies demonstrate various approaches for balancing infection control with osseointegration. Agluna® [429], used clinically, incorporates Ag^+^ into anodized titanium oxide, releasing antimicrobial doses during the postoperative window when infection risk is high. Acusure Ag®, embedding silver into hydroxyapatite plasma-sprayed coatings, extends this principle to bone-contacting surfaces, maintaining osteoconductivity while deterring bacterial adhesion. HyProtect™ [430], an ultrathin polysiloxane plasma–silver coating, has been applied to orthopedic, dental, trauma, and hernia implants, while MicroSilver BG™ microporous formulations expand use to bone cement, sprays, creams, and diabetic foot care dressings [431].

From a design perspective, coatings must provide durable antibacterial coverage while preserving mechanical strength and osseointegration. Coating thickness, porosity, and adhesion must be tailored to implant geometry and anatomical site, as excessive release in soft-tissue zones risks fibroblast toxicity. Standardized reporting of dose-normalized ion release under physiological conditions, along with correlation with infection recurrence and implant survivorship, remains a priority for regulatory acceptance [432].

### 9.4 Dentistry: caries control, restoratives, and oral devices

Randomized clinical trials of nano-silver fluoride (NSF) varnishes demonstrate that high-dose formulations achieve caries arrest rates of ∼72.7 % at six months, outperforming both NSF and saline controls. Silver-modified resins and adhesives further reduce the risk of secondary caries in early clinical and *in vitro* studies, with chitosan-capped AgNPs mitigating the discoloration observed in bulk silver preparations [[433], [434], [435], [436]]. Commercial innovations extend this principle to implants. SilverPlug®, a silver-zeolite polymer sealant, is inserted into implant screw tunnels to reduce the risk of anaerobic colonization and peri-implantitis. Its contact-killing mechanism avoids systemic ion release while preserving soft tissue integration and aesthetic outcomes [437,438].

Oral environments present unique challenges: salivary proteins quickly form coronas that influence Ag^+^ release, while cyclic chewing forces create high mechanical demands. Embedding low-dose silver into resins or adhesives, along with remineralizing fillers, may provide a sustained antimicrobial benefit without staining. Rigorous evaluation under saliva-mimicking conditions and mechanical fatigue testing is essential to advance translation [439].

### 9.5 Regenerative and theranostic platforms: sensing-and-healing dressings

A new frontier integrates silver into theranostic systems that simultaneously diagnose and treat. Composite hydrogels combining AgNPs with human umbilical cord mesenchymal stem cell EVs (hUCMSC-EVs) have demonstrated enhanced nerve regeneration through infection suppression and the delivery of paracrine repair signals. Silver-based SERS substrates enhance functionality for rapid diagnostics, enabling the accurate distinction of Staphylococcus species and methicillin-resistant strains across thousands of spectra. Photothermal silver sulfide (Ag_2_S) quantum dots extend into the near-infrared window, enabling deep-tissue imaging and light-activated antibacterial effects in preclinical models [[440], [441], [442]]. For these multifunctional devices, reporting should include analytical metrics such as detection limits in wound exudate, specificity versus inflammatory noise, and clinically meaningful endpoints, including time to appropriate therapy, wound closure, and long-term functional recovery.

### 9.6 Withdrawn or restricted uses

Not all silver applications have succeeded clinically, and failures provide critical design lessons. The Silzone® mechanical heart valve, marketed with a silver-coated sewing cuff to prevent endocarditis, was recalled in 2000 after the AVERT trial identified increased paravalvular leaks despite reduced infection rates. This failure demonstrated that antimicrobial benefit cannot compensate for compromised hemodynamics or tissue integration [443,444]. Similarly, oral colloidal silver products remain unapproved; in 1999, the U.S. FDA declared that colloidal silver is neither safe nor effective for any condition. Long-term use can cause argyria, an irreversible bluish-gray discoloration resulting from the accumulation of silver in tissue. These instances underscore the importance of regulatory oversight, thorough preclinical testing, and post-market monitoring to ensure that antimicrobial coatings meet the physiological and biomechanical requirements of their targeted sites [445].

### 9.7 Key cross-cutting insights for clinical translation

Across clinical fields, several common principles arise. Materials must be suited to their microenvironment: hydrogels for moist wounds, anti-fouling thin films for airway devices, and mechanically sturdy composites for orthopedic and dental uses. Dosing should be based on release-normalized factors, such as ion flux in chloride- and protein-rich fluids, ζ-potential, hydrodynamic size, and corona composition, rather than just bulk mass loading. Evaluation endpoints also need to be clinically relevant, going beyond microbial kill curves to include surgical site infection rates, pain relief, time to re-epithelialization, ventilator-associated pneumonia rates, implant longevity, and cavity prevention [446]. Low-dose synergy, achieved by combining silver with photothermal, enzymatic, or immune-modulating boosters, provides an alternative approach to reducing cytotoxicity while maintaining effectiveness and following SbD principles. Ultimately, reproducibility and regulatory compliance require the early adoption of high-throughput discovery methods, machine learning optimization, and standardized omics-based testing, all supported by FAIR-compliant data systems [447,448]. These insights not only define the technical logic of effective silver systems but also establish the foundation for regulatory and translational expectations. The journey from laboratory innovation to clinical use ultimately depends on whether these design principles can align with predictable safety profiles, reproducible manufacturing, and compliance with international standards.

## 10 Regulatory, safety, and translational frameworks for silver nanocomposites

The clinical adoption of silver nanocomposites depends not only on their antimicrobial effectiveness but also on their ability to demonstrate safety, immune compatibility, batch-to-batch consistency, and compliance with evolving regulatory standards. In addition to showing pathogen elimination, regulators now require evidence of dose limits, mechanistic safety data, adherence to quality systems, and post-market surveillance [449].

### 10.1 Dose windows and cytotoxicity

Silver's antimicrobial effectiveness stems from Ag^+^ release, but this same process causes oxidative stress in mammalian cells. Cytotoxicity depends heavily on size and dose: smaller AgNPs (around 5–15 nm) produce more ROS and are more toxic than larger particles. At high concentrations, in the tens of μg/mL range, they can disrupt mitochondrial function, reduce glutathione levels, and activate caspases [450]. Defining safe therapeutic windows thus requires normalizing the dose as Ag^+^ flux over time in physiologically relevant fluids rather than as bulk mass loading. SbD strategies reduce toxicity through surface engineering, such as polydopamine, zwitterionic, or phytochemical coatings, which modulate dissolution kinetics and broaden biocompatible ranges [451].

### 10.2 Oxidative stress and immune responses

Exposure to AgNPs induces oxidative stress pathways, activating NF-κB and MAPK cascades and upregulating cytokines such as IL-6, TNF-α, and IL-1β. Excess ROS also triggers mitochondrial dysfunction, DNA damage, and apoptotic or pyroptotic cell death. Mitigation strategies incorporate antioxidants (e.g., curcumin, CeO_2_) or EVs, which inhibit inflammatory signaling while supporting regeneration. The protein corona further influences immune interactions: serum-derived coronas decrease uptake but can still trigger inflammatory cytokines, emphasizing the need for systematic analysis of corona composition, complement activation, and clearance dynamics [450,452].

### 10.3 Regulatory pathways

In the United States, silver-containing medical devices are regulated based on their intended use and the duration of patient contact. Products such as wound dressings, catheters, and dental applications are often reviewed through streamlined pathways, while higher-risk devices or those incorporating drugs may be subject to more extensive evaluation. Recent regulatory updates emphasize the need for alignment with international quality management standards, which require robust design controls, effective supplier oversight, and thorough process validation.

In the European Union, the Medical Device Regulation contains specific provisions for devices that incorporate nanomaterials. Classification depends on the potential for internal exposure, with higher exposure levels leading to stricter scrutiny. Manufacturers must demonstrate appropriate characterization of material properties, dissolution or release behavior, and systemic exposure, and provide evidence that safety margins are respected.

Across both regions, compliance is supported by international standards and technical reports that guide biological evaluation, physicochemical characterization, and stability testing of nanomaterials. References such as the ISO 10993 series, ISO/TR 13014, and OECD test guidelines provide widely accepted frameworks. Increasingly, regulators expect structured and transparent data and documentation that enable consistent review and traceability.

### 10.4 Manufacturing, reproducibility, and GMP compliance

Batch-to-batch reproducibility remains a critical challenge. Slight variations in synthesis conditions, such as pH, reductant feed, or capping agents, can shift Ag^+^ release profiles and biological interactions. Process Analytical Technology (PAT), integrated into microfluidic or continuous flow reactors, enables real-time monitoring and control, while closed-loop platforms combining Bayesian optimization with spectroscopic feedback converge rapidly on target plasmonic and dissolution profiles. Regulatory frameworks require manufacturers to demonstrate lot-to-lot consistency, sterility, and stability after sterilization and aging. Digital twins of synthesis workflows provide a pathway to scale up under Good Manufacturing Practice (GMP) conditions while preserving critical quality attributes, such as hydrodynamic size, Ag^+^ release rate, and coating density.

### 10.5 Post-market surveillance and translational vigilance

**Fig. 11:**
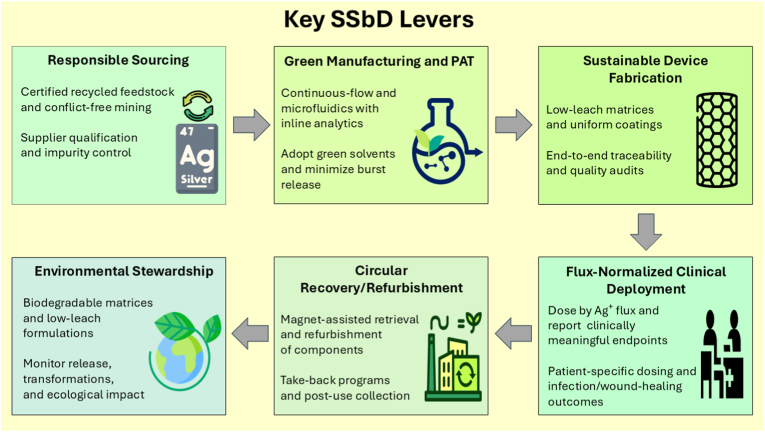
Safe and sustainable by design (SSbD) lifecycle and environmental fate of silver nanocomposites. This diagram outlines the cradle-to-grave lifecycle of silver nanocomposites under SSbD principles, connecting material sourcing, automated production, clinical deployment, and post-market environmental monitoring. Upstream components include the responsible sourcing of silver precursors and PAT-enabled manufacturing, which enables real-time control of composition and ion flux. During clinical use, flux-based dosing ensures therapeutic efficacy while minimizing toxicity. Post-use, materials enter circular recovery pipelines (e.g., scaffold retrieval or recycling) or waste streams (e.g., dressings, implants). The environmental fate is evaluated through a life cycle assessment, considering nanoparticle release into water, soil, or biological waste, and integrating it with post-market surveillance platforms. This SSbD framework aligns with emerging European regulatory expectations, emphasizing both patient safety and ecological stewardship as part of long-term nanomaterial governance.

Translation does not end at approval. Post-market surveillance is essential to identify long-term risks, including the emergence of silver resistance through efflux pumps or metalloresistance genes. Case reports of argyria and the withdrawal of devices such as the Silzone® heart valve underscore the need for vigilance. Environmental sustainability is now embedded in European regulatory expectations through SSbD initiatives, requiring lifecycle assessments that track potential nanoparticle release into wastewater or soil. Clinical-grade silver products must therefore be monitored not only for patient safety but also for ecological footprint [453]. This system-level progression is illustrated in Fig. 11, which outlines the SSbD lifecycle for silver nanocomposites, tracing environmental exposure and sustainability responsibilities from material sourcing and manufacturing through clinical use, post-market recovery, and ecological monitoring.

### 10.6 Section summary

For silver nanocomposites to advance from simple dressings to next-generation implants and theranostic systems, they must meet multiple criteria. Regulators now demand measurable dose windows, mechanistic safety data, ISO 13485-compliant manufacturing, and post-market monitoring. Embedding these translational requirements into early design stages will be essential to unlock the clinical and societal benefits of silver nanocomposites.

## 11 Future directions and transformative visions

Silver nanocomposites are approaching a transition point where their role extends beyond antimicrobial adjuncts into programmable, multifunctional systems. Unlike current devices that rely primarily on passive ion release, future materials will be designed to sense, compute, and adapt within biological and environmental contexts. These directions require integration of nanomaterials engineering, synthetic biology, digital health, and ecological stewardship, positioning silver not just as an additive but also as a responsive, sustainable, and ethically managed component of therapeutic and diagnostic systems. Next-generation dressings and implants will link diagnostic feedback directly to therapy. Stimulus-responsive matrices already demonstrate selective Ag^+^ release under changes in pH or temperature; coupling these with embedded biosensors and wireless communication modules would create closed-loop systems that monitor biomarkers such as protease activity or local pH and autonomously adjust silver dosing. Bluetooth-enabled hydrogels and microprocessor-integrated bandages demonstrate how cyber-physical systems could turn silver composites into active components of digital health networks.

Future antimicrobial strategies must distinguish pathogens from commensals to maintain microbial balance. At sub-inhibitory concentrations, AgNPs can disrupt quorum sensing, reducing virulence pathways without killing entire microbial communities. Incorporating quorum-sensing detection components into silver composites could activate ion release only when pathogenic colonization thresholds are surpassed, enabling microbiome-sensitive therapies for wounds, gut-associated implants, or dental devices [454]. Silver's plasmonic resonance makes it uniquely suited for viral sensing. SERS platforms on Ag substrates have achieved viral detection at extremely low particle counts, pointing toward future protective devices, masks, filters, or implant coatings that not only detect airborne or fluid-borne viruses but also neutralize them *in situ*. Such theranostic constructs could provide simultaneous barrier protection and point-of-care diagnostics [455]. Silver interacts with the immune system in a size- and dose-dependent manner, affecting neutrophil extracellular trap formation and macrophage polarization. Future composites might harness this plasticity by delivering a temporary pro-inflammatory response to eliminate pathogens, then shifting to pro-healing states through co-release of immunomodulators or EVs. Such dual-phase designs could integrate antimicrobial therapies with immune-programming implants [456]. Mechanobiology highlights how stiffness and viscoelasticity regulate cell differentiation and inflammation. Silver composites embedded in degradable hydrogels could be engineered with time-dependent stiffness, softening as healing advances which would allow materials to provide antimicrobial protection during early closure and then guide tissue regeneration through mechanically tuned cues, merging infection control with long-term repair programming [457]. Silver's photothermal and catalytic properties enable external energy harvesting for controlled therapy. Future dressings may harness near-infrared light or alternating magnetic fields to trigger localized ROS generation or Ag^+^ release only when necessary, reducing systemic exposure. Similar innovations include microfluidic patches that produce fresh AgNPs *in situ* from safe precursors, offering “on-demand nanomedicine” ideal for remote or emergency care settings [458].

The convergence of silver composites with flexible electronics creates opportunities for “cognitive care” platforms. Inkjet-printed bandages already incorporate sensors for pH, pressure, and oxygenation; combining these with adaptive silver reservoirs and AI-driven analytics could enable real-time infection prediction, wound staging, and automated adjustments to antimicrobial delivery. Such AI-guided feedback loops would integrate silver into intelligent, personalized therapeutic systems [459]. Additive manufacturing holds promise for the decentralized production of silver composites. 3D-printed scaffolds offer antimicrobial properties, mechanical support, and promote angiogenesis. Extending this method to open-source design files and benchtop microfluidic synthesis could enable clinics in resource-limited areas to create custom dressings and implants, making access more equitable while ensuring reproducibility and quality control in distributed manufacturing systems [460].

As silver nanomedicine scales, recovery and reuse will be critical. Dressings and implants could be designed with magnetizable or chemically extractable phases to enable clinical reclamation, thereby reducing both cost and environmental impact. Upstream, plant- and microbe-mediated syntheses under mild conditions reduce reliance on toxic reagents. These innovations point toward a circular economy model where silver is responsibly sourced, used, and recycled across the product lifecycle [461]. Embedding autonomy and intelligence into silver composites introduces new governance challenges. AI-driven dosing requires transparent, auditable algorithms; decentralized fabrication raises questions of quality assurance and intellectual property; circular-economy designs demand rigorous life-cycle assessments to prevent unintended ecological burdens. Addressing equity in access and regulatory oversight will be as important as engineering multifunctionality if silver nanocomposites are to transition responsibly into mainstream medicine and public health [462]. Silver's future will not be defined by incremental improvements in current wound dressings or coatings, but by its reinvention as a programmable, adaptive material embedded in digital, biological, and ecological networks. By linking sensing, immune programming, mechanobiology, energy harvesting, distributed manufacturing, and circular recovery, silver nanocomposites could emerge as cornerstones of precision medicine and sustainable healthcare. These transformative visions, however, must be accompanied by ethical oversight, interdisciplinary collaboration, and robust frameworks that ensure safety and foster societal trust.

## 12 Conclusion

The progress of silver nanocomposites has reached a stage where engineering innovation alone is no longer sufficient. Future impacts will depend on how precisely the community can integrate reproducibility, sustainability, and predictive control into design and regulation. While current research has demonstrated that nanoscale tuning of size, shape, and surface chemistry significantly influences silver's antimicrobial and catalytic performance, the next challenge lies in making these parameters reproducible across laboratories and manufacturable at scale. Without standardized synthesis and quality control, the field risks being confined to proof-of-concept studies rather than clinically or industrially viable technologies. Linking mechanistic understanding to biological complexity also remains a critical gap. Most studies focus on single-parameter effects, yet cellular, microbial, and environmental interactions operate as dynamic networks. Bridging such divisions will require coupling materials data with biological and omics datasets to develop predictive models that can anticipate ion release, immune modulation, and/or resistance before *in vivo* testing. Such system-level approaches will transform silver research from empirical observation to mechanistic prediction.

Sustainability must evolve from an afterthought to a design principle. Biogenic and recyclable formulations should be evaluated not only for green synthesis but for their full life-cycle impacts, including silver recovery, waste minimization, and long-term environmental persistence. Integrating circular economic concepts with regulatory frameworks can ensure that innovation aligns with public trust and ecological responsibility. Most importantly, the field must move toward ethical transparency and interdisciplinary governance. As silver materials become autonomous and data-connected, questions of safety, data integrity, and equity of access will become as critical as antimicrobial efficacy. Establishing open databases, reproducible protocols, and globally harmonized standards will be crucial to sustaining credibility and accelerating translation. In this perspective, the ultimate value of silver nanocomposites lies not in any single clinical success but in setting a model for responsible nanotechnology. By uniting precise engineering, predictive analytics, and sustainable ethics, silver nanocomposites can redefine what it means for a material to be both functional and accountable, advancing not only biomedical innovation but also the culture of reproducible, safe, and socially conscious science.

## CRediT authorship contribution statement

**Eileen Tabrizi:** Writing – review & editing, Writing – original draft, Visualization, Resources, Conceptualization. **Bingyun Li:** Writing – review & editing, Visualization, Supervision, Resources, Project administration, Methodology, Funding acquisition, Formal analysis, Conceptualization.

## Declaration of competing interest

The authors declare that they have no known competing financial interests or personal relationships that could have appeared to influence the work reported in this paper.

## Data Availability

No data was used for the research described in the article.
